# Cannabidiol does not cause DNA double-strand breaks in a human liver-derived cell model

**DOI:** 10.1186/s42238-025-00365-w

**Published:** 2025-12-12

**Authors:** Romano Weiss, Victoria Liedtke, Stefan Rödiger

**Affiliations:** Faculty 2 - Environment and Natural Sciences, Brandenburg University of Technology Cottbus-Senftenberg, Senftenberg, Brandenburg 01968 Germany

**Keywords:** Cannabidiol, DNA damage, Proliferation, CAMP, HepG2

## Abstract

**Background:**

Cannabidiol (CBD) is a non-psychoactive cannabinoid with potential therapeutic applications, including anti-inflammatory, analgesic, and anticancer effects. However, experts raised concerns about its potential to induce DNA damage and chromosomal aberrations at low concentrations. Notably, these studies used liver cell lines, which may not fully reflect the metabolic processing of CBD, potentially limiting the generalizability of their findings. This study investigated the short time effects of CBD on DNA double-strand breaks (DSBs) and proliferation in the human liver-derived cell line HepG2.

**Methods:**

HepG2 cells were treated with CBD (5 – 50 $\mu M$, 3 - 72h incubation). To investigate potential imbalances in the expression of cannabinoid receptors 1 and 2 (CB1 / CB2) within HepG2 cells, we examined their expression using Western blot analysis. We hypothesized that such an imbalance could be associated with pathogenic processes. Double-strand breaks were then detected (5 $\mu M$ Etoposide (ETP) served as positive control) via indirect immunofluorescence analysis using $\gamma$ H2AX and 53BP1 antibodies, followed by quantification of DSB foci.

**Results:**

Expression of CB2 but not CB1 was downregulated by 30 % in HepG2 cells after exposure to 5 $\mu M$ CBD (24h incubation; $p<$0.05) and 70 % downregulated after exposure to 50 $\mu M$ CBD (24h incubation; $p<$0.01). This effect was dose-dependent. Whilst ETP induced dose dependent DSBs, we could not confirm findings by others that CBD significantly increases the number of $\gamma$ H2AX and 53BP1 foci between 5 $\mu M$ and 50 $\mu M$ (3h incubation; $p<$0.05).

**Conclusion:**

In our model, CBD stimulated the cells, as confirmed by modulation of CB2 expression as well as changes in intracellular cAMP. Our results show that CBD in ranges between 5 $\mu M$ to 50 $\mu M$ does not significantly increase the amount of DNA double strand breaks in HepG2 cells compared to the control. However, we did observe a significant reduction in cell proliferation and a significant increase in intracellular cAMP levels following CBD treatment.

**Supplementary Information:**

The online version contains supplementary material available at 10.1186/s42238-025-00365-w.

## Background

The plant Cannabis sativa has received considerable attention, particularly for its bioactive compounds known as cannabinoids (Liu et al. 2022). Among these, cannabidiol (CBD) has emerged as a non-psychoactive cannabinoid with considerable therapeutic potential, for example in oncology (Heider et al. 2022). Unlike $\Delta$9-tetrahydrocannabinol (THC), which has psychoactive effects, mediated through CB1 receptor activation (Izzo And Camilleri 2009), CBD interacts with a wide range of molecular targets, including cannabinoid receptors (CB1 and CB2), transient receptor potential (TRP) channels, and nuclear receptors such as peroxisome proliferator-activated receptors (PPARs) (Heider et al. 2022). These interactions suggest a diverse mechanism by which CBD may exert anti-cancer effects. Preclinical studies highlighted mechanisms by which CBD may affect cancer biology. CBD has been shown to induce apoptosis in various cancer cell lines, including breast (Shrivastava et al. 2011), prostate (O’Reilly et al. 2023) and glioblastoma (Gross et al. 2021), by modulating key apoptotic regulators such as p53 (O’Reilly et al. 2023), Bax (Shrivastava et al. 2011) and caspases (Gross et al. 2021), while downregulating anti-apoptotic proteins such as Bcl-2 (Shrivastava et al. 2011). CBD has been shown to inhibit angiogenesis and metastasis by targeting pathways critical for tumor growth and dissemination, such as the vascular endothelial growth factor (VEGF) pathway (Blázquez et al. 2004) and matrix metalloproteinases (MMPs) (Gęgotek et al. 2021). Additionally, CBD can modulate the tumor microenvironment by exerting anti-inflammatory (Lowin et al. 2020) and immunomodulatory effects, which may contribute to its ability to suppress tumor progression and metastasis. CBD sensitizes cancer cells to chemotherapeutic agents like cisplatin by modulating drug resistance pathways, highlighting its potential to enhance the efficacy of conventional cancer treatments. One study demonstrated that treatment of HepG2 and MDA-MB-231 cancer cells with 3 $\mu M$ and 5 $\mu M$ CBD for 24 h, followed by exposure to 100 $\mu M$ cisplatin for an additional 24 h, resulted in a synergistic effect, with a cell toxicity increase exceeding 100 % (O’Brien 2022). In glioblastoma, CBD increases the cytotoxic effects of temozolomide, a standard chemotherapeutic agent, by promoting oxidative stress and endoplasmic reticulum (ER) stress, leading to increased cancer cell death (Soroceanu et al. 2022). The anti-inflammatory and antioxidant properties of CBD might be beneficial in reducing the adverse effects of chemotherapy, including neuropathy, nausea and oxidative damage, and could lead to an improvement in the quality of life of patients undergoing such treatment. CBD’s modulation of cytokine production and reduction of reactive oxygen species (ROS) (Pereira et al. 2021) may help to reduce the inflammatory response that often accompanies both cancer and its treatment, thereby further underscoring its potential as an adjunct therapy. Research also indicates harmful effects of CBD on the DNA integrity, especially regarding DNA damage and chromosomal aberrations (Li et al. 2024; Paganoet al. 2024; Russo et al. 2019). Studies have demonstrated the cytotoxic effects of CBD on human gingival fibroblasts and oral keratinocytes, particularly at higher concentrations (50 $\mu M$ and 100 $\mu M$), where significant reductions in cell viability were observed (Paganoet al. 2024). Conversely, lower concentrations (25 $\mu M$ and below) appeared to enhance cell viability in human colorectal cancer cells HT-29 (Sainz-Cort et al. 2020), suggesting a potential dose-dependent response. In contrast, some studies highlight CBD’s potential genotoxicity and carcinogenicity. Using a single-cell gel electrophoresis assay (SCGE, also known as the comet assay), these studies demonstrate that CBD can cause DNA damage and chromosomal aberrations in human cells at relatively low concentrations (0.66 to 54 $\mu M$) (Russo et al. 2019). There are several types of DNA damage, including single-strand breaks (SSBs), double-strand breaks (DSBs), oxidative lesions (e.g., 8-hydroxy-2’-deoxyguanosine, or 8-OHdG), and chromosomal rearrangements such as deletions, translocations, or point mutations. DSBs are particularly harmful, as they can lead to genomic instability and increased cancer risk. There are assays to quantify DNA damage (Reddig et al. 2018; Ruhe et al. 2019, 2019). While the comet assay can be useful for identifying DNA damage, it has various limitations that make it less than ideal for comprehensive assessment. One major drawback is its lack of specificity in distinguishing between different types and severities of DNA lesions, which may lead to overestimation of double-strand breaks (DSBs). Furthermore, factors such as cell cycle stage and chromatin structure can impact the assay’s sensitivity, resulting in inconsistent results across various cell types or experimental conditions (Hosking et al. 2024). Gamma-H2AX ($\gamma$H2AX), a phosphorylated form of histone H2AX, and 53PB1 (p53 binding protein 1) are highly specific biomarkers for DSBs and are detectable via immunofluorescence or automated foci counting methods (Schneider et al. 2019; Weiss et al. 2022). This offers an alternative to the comet assay for quantifying DSBs with greater precision, but other forms of DNA damage, such as oxidative stress-induced lesions (e.g., 8-OHdG) or point mutations, require complementary assays like immunohistochemistry or fluorescence *in situ* hybridization (FISH) (Ruhe et al. 2019; Ivashkevich et al. 2012).

However, H2AX phosphorylation can also be caused by factors other than direct DNA double-strand breaks, including replication stress, chromatin remodeling, cell cycle-regulated events, apoptosis, single-strand breaks with subsequent ATR activation and cellular repair mechanisms (Bonner et al. 2008; Mah et al. 2010; Gagou et al. 2010; Baure et al. 2008; Ichijima et al. 2005; Solier And Pommier 2014; Schütz et al. 2021). Therefore, simultaneous detection of other DNA double-strand break markers such as 53BP1 is advisable (Popp et al. 2017).

We used a HepG2 liver cell model to investigate CBD mediated DNA damaging effect on protein and cellular level, complemented with the analysis of the metabolic state by cAMP measurement. HepG2 cells have gained recognition as a reliable model for examining the potential DNA-damaging impacts of cannabidiol (CBD). This is because they originate from human hepatocellular carcinoma, and are frequently utilized in evaluating drug metabolism and liver toxicity. In the liver, CBD undergoes significant phase I and II metabolic processes, making HepG2 cells a good choice for such studies (Arzumanian et al. 2021). We analyzed the dose-dependent expression of proteins involved in metabolism, DNA repair, apoptosis, and CBD receptor expression. Although we observed dose-dependent biological effects of CBD, we were unable to demonstrate that CBD at concentrations between 5 and 50 $\mu M$ induces DNA double-strand breaks during an exposure time of 3 h. On the contrary, our observations indicate a slight reduction in the number of DNA double strand breaks. This observation was not dose-dependent for this concentration range.

## Methods

### Cell culture

HepG2 (ATCC® HB-8065™) cells were cultured in Dulbecco’s modified Eagle’s medium Nutrient Mixture F-12 (DMEM/F12) supplemented with 10 % fetal calf serum (FCS), 2 *mM* L-glutamine, and 100 U mL-1 penicillin/streptomycin at 37 $^{\circ }$C in a 5 % $CO_2$ environment. Cells were routinely subcultured twice a week.

### Cannabidiol handling

200 *mM* Cannabidiol (NMI Australia; NMID512B) were diluted in pure ethanol and stored at 4 $^{\circ }$C until usage (stock solution). Working solutions were created right before usage by mixing the created stock solution with culture medium (0 - 50 $\mu M$ CBD). Ethanol was added to achieve a final concentration of 0.05 %.

### Western blot analysis

#### Sample preparation

HepG2 cells ($1*10^6$) were seeded in six-well plates as described in paragraph Cell culture. After a 24-hour incubation period with CBD (0 - 50 $\mu M$), the cells were washed with phosphate-buffered saline (PBS), and 50 $\mu L$ of 2$\times$ Laemmli buffer was added to each well. The cells were lysed by incubating the contents for an appropriate duration. The lysate was harvested and sonicated if necessary. Samples were heated at 95 $^{\circ }$C for five minutes and then centrifuged at 13,000 revolutions per minute (rpm) for five minutes. Protein content was determined using the RotiQuant kit in a 96-well format, following the manufacturer’s instructions. After protein quantification, 5 % (v/v) 2-mercaptoethanol was added to each sample.

#### SDS-PAGE

Sodium dodecyl sulfate-polyacrylamide gel electrophoresis (SDS-PAGE) was performed using a 5 % stacking and 12 % resolving gel. 30 $\mu g$ per lane were loaded; 2–4 $\mu L$ pre-stained PAGE Ruler Plus (Thermo Scientific) were used as molecular weight markers. The gel was run at 50 V for 45 min in the stacking gel, followed by running at 120 V for 90 min. The finished gel was stained using Coomassie Brilliant Blue solution for 15 min on a rocking shaker. Subsequently, the gel was destained (10 % acetic acid and 20 % ethanol) in a shaking incubator at room temperature (RT).

#### Western blotting

Before transfer, the SDS-PAGE gel was equilibrated in the transfer buffer for 5 minutes. A polyvinylidene difluoride (PVDF) membrane was activated with methanol for 20 seconds and then equilibrated in the transfer buffer for 2 minutes. Filter papers (ten per gel) were soaked in a transfer buffer, and western blotting was performed at 50 mA per $cm^2$ of gel for 2 h. To confirm successful protein transfer, the membrane was stained with Ponceau S by washing it with TBST for 5 minutes, incubating it in Ponceau S solution for 5 minutes, and then rinsing it with distilled water until protein bands were clearly visible. To remove the Ponceau S stain, the membrane was washed with TBST for 5 minutes and then blocked with 5 % non-fat dried milk in TBST for 1 hour on a rocking shaker. In parallel the gel was stained with coomassie brilliant-Blue to visualize the amount of total protein loaded (see section SDS-PAGE ). The membrane was incubated with an solution (antibody diluted in 2 % non-fat dried milk in TBST) containing the primary antibodies against LEDGF/p75((Bethyl laboratories, Cat# A300-847A, RRID: AB 609466, 1:1000 dilution), CB-1 (Santa Cruz Biotechnology, Dallas, USA, Cat# sc-293419, RRID: N/A, 1:1000 dilution), CB-2 (Santa Cruz Biotechnology, Dallas, USA, Cat# sc-293188, RRID: N/A), GAPDH (Cell Signaling, Technology, Massachusetts, USA, Cat# 2118, RRID: AB 561053, 1: 20.000) and Caspase-3 (Cell Signaling technology, Messacchusetty, USA, Cat# 9662, RRID: AB 331439, 1:1000) for 1 hour at room temperature. After three 5-minute washes with TBST, the membrane was incubated with the secondary antibody solution (horseradish peroxidase-coupled secondary antibody in 5 % non-fat dried milk in TBST) for 1–2 h at room temperature. Following three more 5-minute washes with TBST, the membrane was incubated with enhanced chemiluminescence (ECL) solution for 1–2 minutes before signal detection using a Lumilimager (Roche). Exposure times ranged from 1 second to 15 minutes, depending on signal intensity. Based on recent literature, the expected molecular weight for CB2 is 52/55 kDa, corresponding to a receptor doublet (Zhang et al. 2007). Band intensity was analyzed using ImageJ by using the whole protein amount as a reference.

### Viability and toxicity assays

Cells were seeded at $5*10^3$ cells per well into multiple 96-well plates (Th. Geyer, Renningen, Germany) and incubated for 24 - 96 h at 37 $^{\circ }$C and 5 % $CO_2$. To analyze possible toxic effects of CBD, cells were allowed to attach for 24 h after seeding and incubated with increasing concentrations of CBD (0 - 50 $\mu M$) for an additional 24 - 72 h. To determine the accuracy of cell seeding, one plate was used as a 0 h control. Proliferation and cytotoxicity were analyzed using the automated multispectral fluorescence microscopy platform VideoScan (Rödiger et al. 2012). Cells stained with PI/Hoechst (Hoechst for nuclei; PI for apoptotic cells) were imaged at an exposure time of 0.5–1 seconds. Each well in a 96-well plate was divided into 12 subregions, imaged sequentially at 10$\times$ magnification, and reconstructed as a single image. The number of viable cells was calculated by subtracting PI-positive (apoptotic) cells from the total Hoechst-positive (nucleated) cells. Images were processed using the scikit-image library (v.0.17.2) by converting to grayscale. Regions outside the analyzed well were masked based on average intensity to avoid interference during detection. Median filtering (4 $\times$ 4 neighborhood; skimage.filters.median) was applied to reduce noise. Cell counts per image were determined using Laplacian of Gaussian Blob Detection. Dead cells were excluded by subtracting, PI-positive cells from Hoechst-positive cell counts.

### Immunofluorescence

Cells were seeded at a density of $1*10^4$ cells per well in their respective growth medium (see the Cell Culture section) on 8-well slides and incubated for 24 h at 37 $^{\circ }$C with 5 % $CO_2$. The medium was then aspirated and cells were incubated with increasing concentrations of CBD (0 - 50 $\mu M$; $5\mu M$ ETP served as positive control) in fresh culture medium (with 0.05% ethanol) for an additional 3 h. Following CBD exposure, the medium was removed, and the cells were fixed with 4 % formaldehyde for 15 minutes at RT. The wells were then washed with phosphate-buffered saline (PBS; 140 mM NaCl, 2.7 mM KCl, 1 mM $Na_2HPO_4$ · 2 $H_2O$, and 2 mM $KH_2PO_4$, pH 7.4) and then blocked and permeabilized with 50 $\mu L$ of blocking/permeabilization buffer per well (PBS, 5 % BSA, 0.3 % Triton X-100) for 30 minutes at RT. After removing the blocking buffer, the primary antibodies anti-$\gamma$H2AX (Merck Millipore, Massachusetts, USA: Cat# 05–636-I, RRID: AB 2755003, 1:100 dilution) and 53BP1 (Novus Biologicals, Colorado, USA, Cat# NB100-304SS, RRID: AB 920462; 1:1500 dilution) were added to the slides, within the blocking/permeabilization buffer. The slides were incubated for 60 minutes at RT, followed by three rinses with PBS.

Next, 25 $\mu L$ per well of the secondary antibody solution (anti-mouse IgG (Abcam, Cambridge, United Kingdom, Cat# ab150115, RRID: AB 268794, 1:150 dilution), anti-rabbit IgG (Abcam, Cambridge, United Kingdom, Cat# ab150077, RRID: AB 263035, 1:800 dilution)) along with 0.1 $\mu g * mL^{-1}$ of 4,6-diamidino-2-phenylindole (DAPI) was added to each well. After a one-hour incubation in darkness at RT and another round of PBS rinsing, a drop of mounting medium (abberior Instruments GmbH, Heidelberg, Germany, abberior Mount, Solid Antifade) was applied to each well, followed by the placement of a coverslip. Images were captured after a 24-hour hardening period at RT. An area encompassing approximately 25,500 $\mu m^2$ around the center of each well, containing between 100–400 nuclei, was imaged. Slides not immediately utilized were stored in darkness at 4 $^{\circ }$C. The 53BP1 and $\gamma$H2AX foci count were determined using our NucDetect software with manual count adjustments.

### cAMP assay

Promega’s cAMP-Glo™ assay (cAMP-Glo™ Assay (V1501), Promega, Wisconsin, USA) was used to monitor the response to the CBD treatment on the G protein-coupled receptors (GPCRs). The assay is based on the principle that cAMP (cyclic adenosine monophosphate) stimulates the activity of PKA (protein kinase A holoenzyme), which decreases the available ATP (adenosine triphosphate) and reduces luminescence in a coupled luciferase reaction. Preparation of cells was performed as follows: $5*10^4$ cells per mL were seeded into multiple wells of a 96-well plate and allowed to attach for 24 h at 37 $^{\circ }$C, 5 % $CO_2$. CBD treatment was applied for 3 h at concentrations of 0–50 $\mu M$, as well as a no treatment control, a solvent control and a control based on 5 $\mu M$ etoposide (ETP). Analysis of cAMP level was performed following the manufacturer’s description.

### Statistical data analysis

To analyse the difference in protein expression, cAMP concentration and cellular proliferation between the test groups, the Kruskal-Wallis-Test was used. To assess the significance of differences in foci counts between test groups, a permutation test was performed (Cramér–von Mises criterion, $$scipy.stats.cramervonmises\_2samp$$) as test statistic. The following parameters were used: observed values for group A (x), observed values for group B (y), method = ‘auto’, axis = 0, $$nan\_policy = 'propagate'$$, and keepdims = False (scipy v.1.15.2). The permutation test was conducted using scipy.stats.permutation_test (parameters: data containing both negative control and test group values, statistic: $$scipy.stats.cramervonmises\_2samp$$, $$permutation\_type: `independent'$$, $$n\_resamples: 9999$$, $$alternative: `two-sided'$$, *axis* : 0, *rng* : 42 (scipy v.1.15.2). The following thresholds were used to determine statistical significance: $$p> 0.05 = \text {not significant (n.s.)}$$, $$p < 0.05 = *$$, $$p < 0.01 = **$$, $$p < 0.005 = ***$$.

## Results

### HepG2 cells show concentration-dependent decrease in viability markers after CBD treatment

**Fig. 1:**
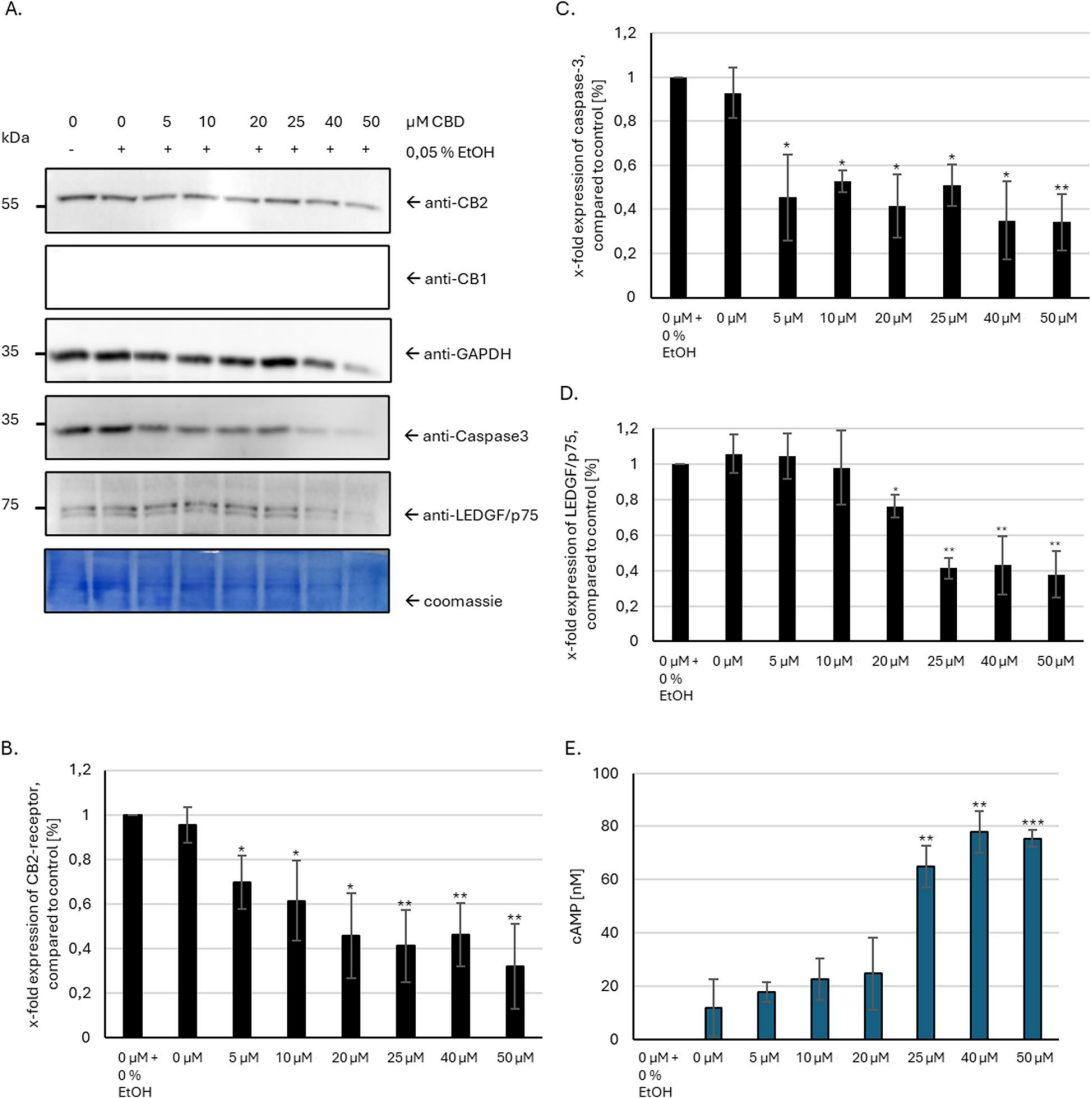
HepG2 cells show a concentration-dependent reduced expression of metabolic, DNA repair and uncleaved apoptotic proteins. **A** Immunoblot using antibodies against cannabinoid-receptor (CB) 1, CB 2, caspase-3 and LEDGF. Cells were incubated for 24 h with 5 - 50 $$\mu M$$ CBD. GAPDH was used as loading control and the whole protein amount was verified using Coomassie brilliant blue staining. No expression of CB1 was observed. Expression of CB2 in HepG2 cells was confirmed by observing a 52/55 kDA protein band (receptor doublet). **B - D** Quantitative analysis of CB2, caspase-3 and LEDGF/p75 expression ($$n = 3$$). **E** Analysis of dose-dependent cAMP concentration after increasing concentrations of CBD. $$p < 0.05 = *$$, $$p < 0.01 = **$$, $$p < 0.005 = ***$$

To elucidate the mechanism of action, discern off-target effects, and ensure selectivity and specificity of CBD treatment in hepatocellular carcinoma, we analyzed the expression of CB1 and CB2 receptors in HepG2 cells. As shown in immunoblot analysis (Fig. 1A) HepG2 cells only express the CB2-receptor subtype, but not CB1. The expression pattern of all proteins was as expected with 55 kDa for CB2 receptor doublet, 35 kDa for GAPDH (Glyceraldehyde 3-phosphate dehydrogenase), 32 kDa for caspase-3 and 75 kDa for LEDGF expression. In addition, protein expression of GAPDH, caspase-3 and LEDGF/p75 was decreased with higher doses of CBD. Quantitative analysis of band intensity (Fig. 1B - D) confirmed the first impression and showed a significantly reduced expression of all analyzed proteins after induction of CBD treatment.

### CBD treatment leads to modified GPCR

**Fig. 2:**
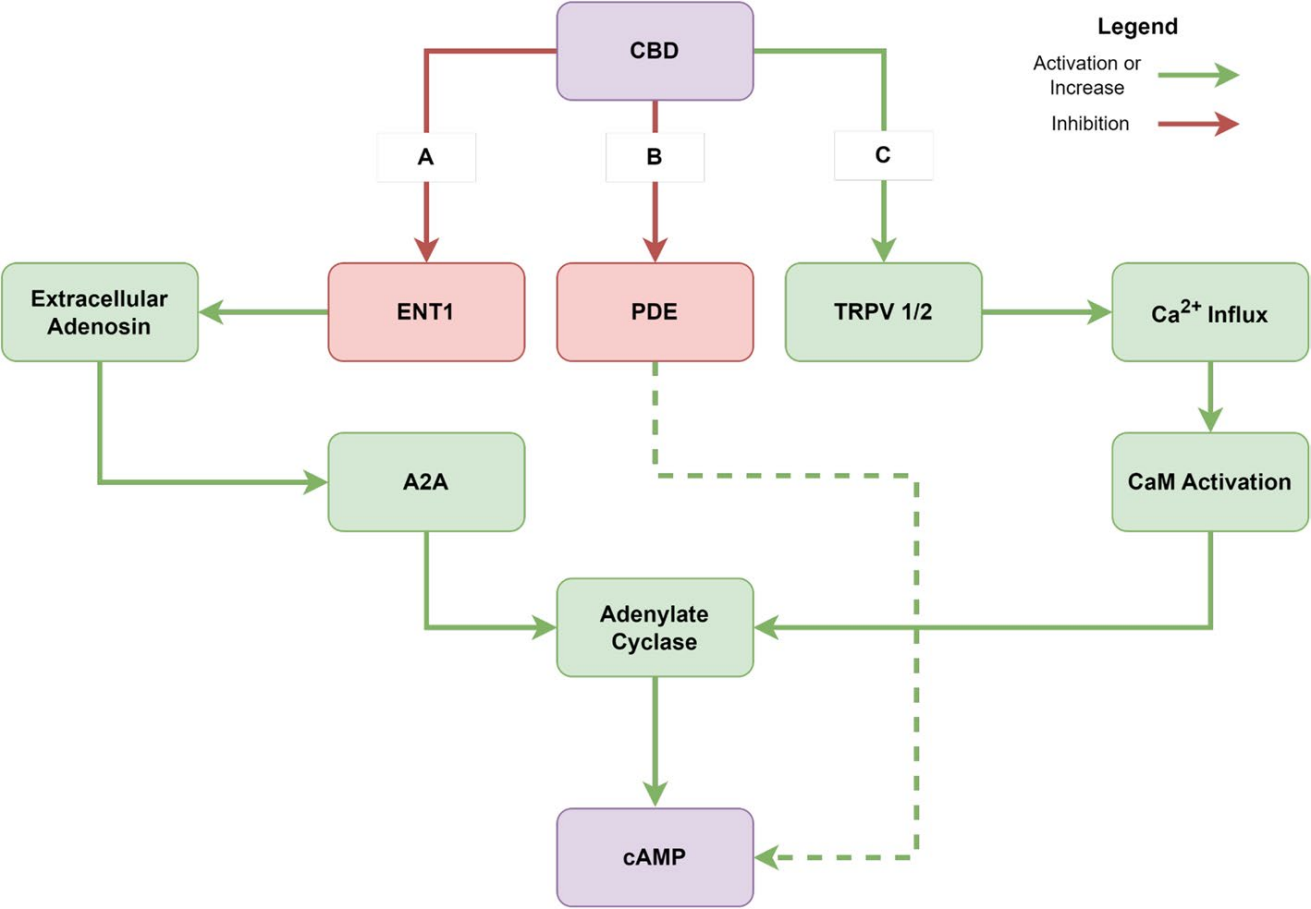
Potential pathways in which Cannabidiol (CBD) could increase intracellular cAMP concentrations. **A** Inhibition of Equilibrative nucleoside transporter (ENT) 1: Inhibition of ENT1 increases the concentration of extracellular adenosine. This activates the Adenosine A2A receptor, a $G_{\alpha S}$ coupled GPCR, which in turn activates the adenylate cyclase. **B** Inhibition of Phosphodiesterases (PDE): PDEs such as PDE4 are directly responsible for the hydrolysation of cAMP in the cytosol. Inhibition of such PDEs would indirectly increase cytosolic cAMP concentrations. **C** Activation of Transient Receptor Potential Vanilloid (TRPV) 1 and 2: Activation of TRPV 1/2 would lead to calcium ion influx, activating calmodulin (CaM), in turn activating calmodulin dependent adenylate cyclases

CB1 and CB2 are G-protein coupled receptors (GPCRs) that utilize inhibitory G0 and G1 proteins for signal transduction. To assess whether CBD modulates GPCR-based signaling and to gain insights into its broader effects on cellular metabolism, we employed cAMP concentration analysis as a versatile indicator. We found that CBD concentrations exceeding 20 $\mu M$ significantly elevate intracellular cAMP levels significantly compared to the control group (Fig. 1E). As both cannabinoid receptors are canonically coupled to inhibitory G proteins, the observed increase in intracellular cAMP concentration has to be mediated by other molecular mechanisms (Fig. 2).

### Cellular proliferation of HepG2 cells is negatively affected by CBD treatment

**Fig. 3:**
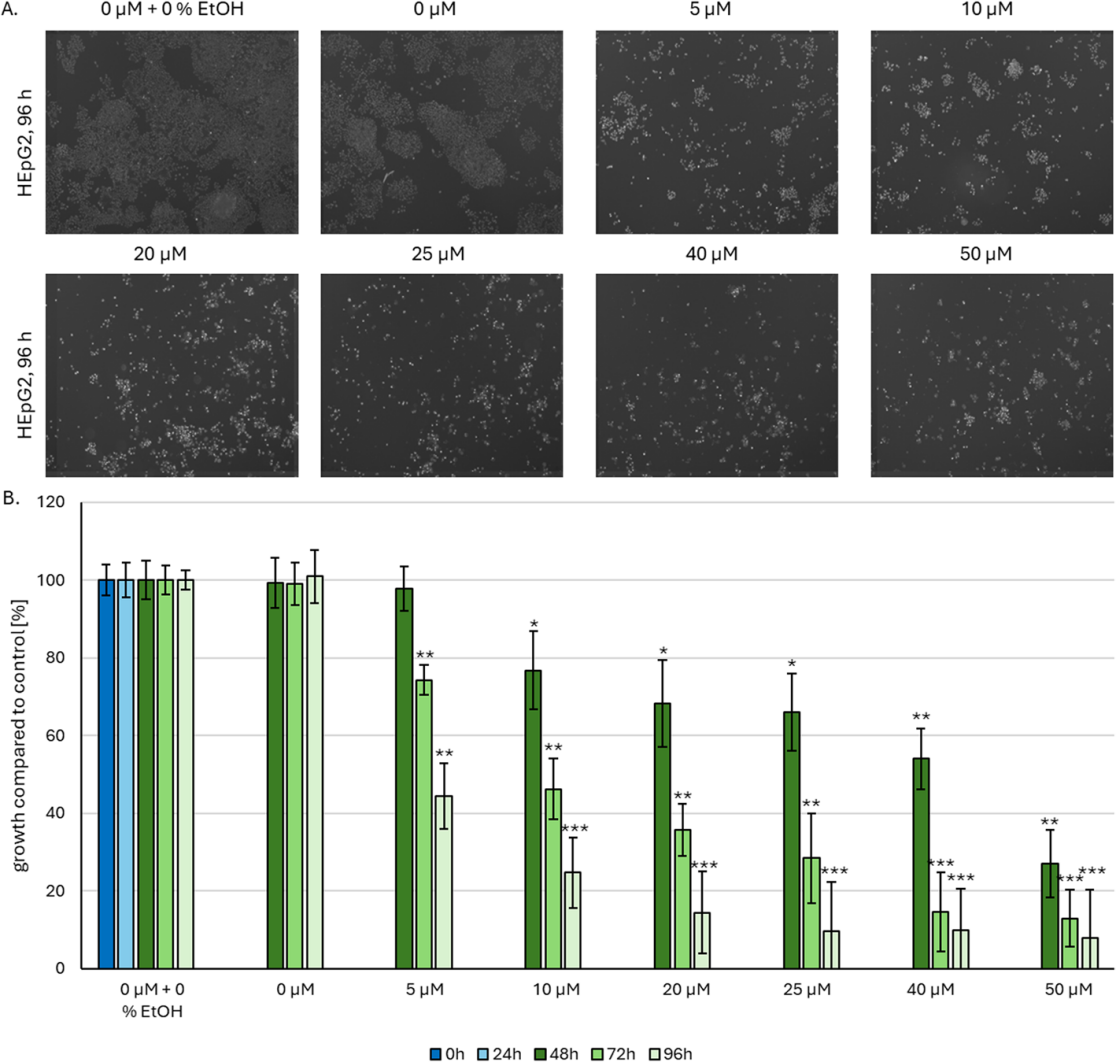
CBD treatment shows dose-dependent influence on cellular proliferation. **A** Fluorescence pictures of HepG2 cell nuclei (grey), Hoechst-stained after 72 h CBD-treatment and a total of 96 h growth. Non-treatment control and solvent control were included. **B** Analysis of cellular proliferation was performed using fluorescence microscopy ($$n = 3$$). Images were taken directly after seeding (0 h, after the cells settled on the bottom of the well) and after 24 h incubation. After that, the different incubation media were added (0–50 $$\mu M$$ CBD with 0.05 % EtOH) and further images were taken after 48 h (24 h CBD incubation), 72 h (48 h CBD incubation) and 96 h (72 h CBD incubation). Cells were incubated at 37 $$^{\circ }$$C in a 5 % $$CO_2$$ environment. $$p < 0.05 = *$$, $$p < 0.01 = **$$, $$p < 0.005 = ***$$

CBD treatment at concentrations of 5–50 $\mu M$ significantly ($p < 0.005$) reduced proliferation of HepG2 cells. As shown in Fig. 3A, confocal images following Hoechst staining demonstrated a dose-dependent decrease in the number of viable cells. Cells were stained with Hoechst (nuclei) and propidium iodide (PI; for apoptotic cells). Viability was calculated by subtracting the number of PI-positive (apoptotic) cells from the total Hoechst-stained (nuclei) cell population. Proliferation rates in control groups—untreated and solvent controls (0.05 % ethanol)—remained constant at approximately 100 % (Fig. 3B). However, CBD treatment significantly decreased cell viability as early as 24 h post-treatment. At 5 $\mu M$ CBD, cell count initially declined to 97 %, further decreasing to 74 % after 48 h and reaching 44.5 % following 72 h of exposure. A similar dose-dependent reduction in viability was observed across all concentrations tested (10–50 $\mu M$). CBD treatment at concentrations of 5–50 $\mu M$ profoundly impaired HepG2 cell proliferation, as evidenced by the dose-dependent decrease in viable cell numbers visible in confocal images following Hoechst staining (Fig. 3A). To quantify viability, cells were co-stained with Hoechst and PI, with viable cells determined by subtracting PI-positive cells from the total Hoechst-positive population. Proliferation rates in both untreated controls and solvent-treated groups (0.5 % ethanol) remained stable at approximately 100 % throughout the experiment (Fig. 3B). Notably, CBD treatment induced a significant reduction in viability within 24 h: 5 $\mu M$ CBD reduced cell count to 97 %, which further declined to 74 % after 48 h and stabilized at 44.5 % following 72 h of exposure. This dose-dependent decrease in viability was consistently observed across concentrations of 10–50 $\mu M$ (Fig. 3B).

### CBD reduces 53BP1 and $\gamma$H2AX foci formation in HepG2 cells

**Fig. 4:**
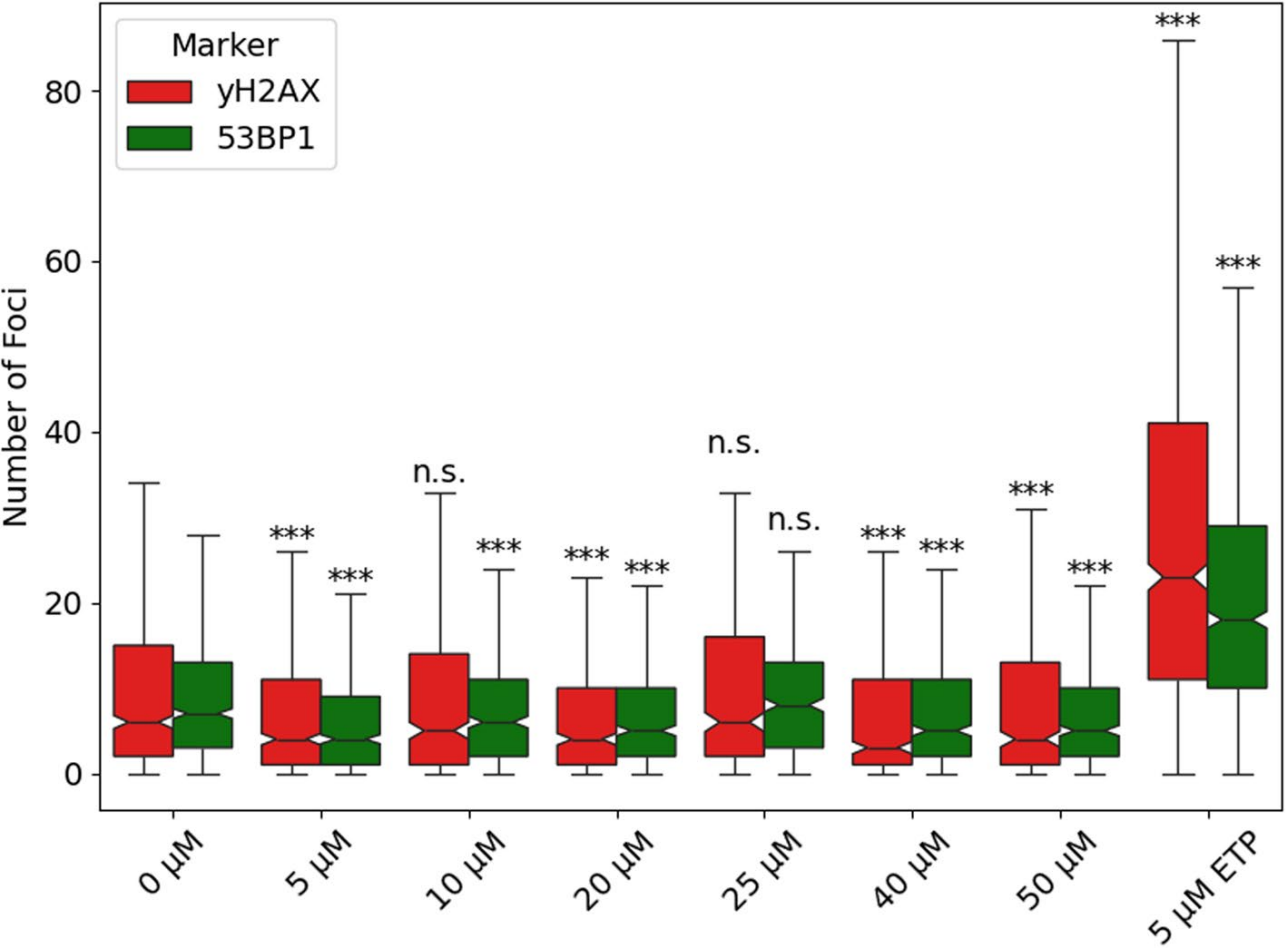
Analysis of indirect immunofluorescence images with $$\gamma$$H2AX and 53BP1 foci of HepG2 cells incubated with CBD (5 - 50 $$\mu M$$ in culture medium with 0.05 % ethanol as solvent; 5 $$\mu M$$ Etoposide (ETP) served as positive control). Cells were incubated for 3 h at 37 $$^{\circ }$$C in a 5 % $$CO_2$$ environment and directly fixated afterwards. Significance was tested against the negative control (0 $$\mu M$$) $$p \ge 0.05 = \text {not significant (n.s.)}$$, $$p < 0.05 = *$$, $$p < 0.01 = **$$, $$p < 0.005 = ***$$

The influence of CBD on DNA damage in HepG2 cells was investigated by treating the cells with varying CBD concentrations (5 - 50 $\mu M$). Etoposide at a concentration of 5 $\mu M$ served as the positive control. Indirect immunofluorescence imaging via laser microscopy revealed the formation of 53BP1 and $\gamma$H2AX foci, which are established DSB markers. To analyze the differences in foci distribution, the empirical cumulative distribution function (ECDF) for 53BP1 and $\gamma$H2AX foci was calculated for each treatment group and compared to the negative control (0 $\mu M$) using the Cramér–von Mises criterion. The results demonstrated a significant reduction in both 53BP1 and $\gamma$H2AX foci distribution for most CBD-treated groups, except for the 10 $\mu M$ and 25 $\mu M$ CBD groups for 53BP1 and $\gamma$H2AX, respectively. However, no dose-dependent effect was observed across the tested concentration range (Fig. 4). Although CBD treatment significantly altered the ECDFs of both 53BP1 and $\gamma$H2AX foci in HepG2 cells ($p <0.005$), the average reduction in foci per nucleus was relatively small, approximately 2 and 1.62 foci for 53BP1 and $\gamma$H2AX, respectively. Despite the statistical significance, no clear dose-effect dependency was evident (Table 1).

**Table 1:** Statistical comparison between CBD test groups. Differences between groups were tested as described in paragraph Statistical data analysis

Marker	Test group	5	10	20	25	40	50
$\gamma$H2AX	5						
	10	n.s.					
	20	n.s.	n.s.				
	25	**	n.s.	***			
	40	n.s.	*	n.s.	***		
	50	n.s.	n.s.	n.s.	***	n.s.	
53BP1	5						
	10	***					
	20	n.s.	*				
	25	***	*	***			
	40	***	n.s.	n.s.	***		
	50	n.s.	*	*	***	n.s.	

*p* ≥ 0.05 = not significant (n.s.), *p* < 0.05 = ∗, *p* < 0.01 = ∗∗, *p* < 0.005 = ∗ ∗ ∗

## Discussion

Our investigation into the effects of cannabidiol (CBD) on DNA integrity and cellular mechanisms in HepG2 cells reveals insights that could reshape our understanding of CBD’s therapeutic potential and safety profile.

### CBD receptor expression and cell proliferation

The observed concentration-dependent decrease in CBD receptor expression in HepG2 cells suggests a potential adaptive response to increasing CBD levels. This downregulation of receptors may be a cellular mechanism to mitigate the effects of high CBD concentrations, similar to the desensitization observed with prolonged exposure to CB1 receptor agonists (Kendall And Yudowski 2017). The decrease in HepG2 cell proliferation at CBD concentrations of 5 $\mu M$ and above, with significant reductions at 10–50 $\mu M$, aligns with previous research demonstrating CBD’s anti-proliferative effects on cancer cells (Mashabela And Kappo 2024; Štern et al. 2024; Billi et al. 2025; Shangguan et al. 2021; Chen et al. 2024). The anti-proliferative effect of CBD on HepG2 cells is consistent with findings in other cancer cell lines. For instance, CBD has been shown to induce concentration-dependent cell death in breast cancer cell lines through the activation of the intrinsic apoptotic pathway (Mashabela And Kappo 2024). In glioma cells, CBD treatment resulted in a reduction of tumor size and metastatic nodules in animal models (Mashabela And Kappo 2024). The lack of effect in vector control cells suggests that the anti-proliferative action is specific to CBD treatment and may involve cannabinoid receptor-mediated pathways.

### CBD modulates cAMP levels and cellular metabolism in HepG2 cells

The increase in cAMP levels with rising CBD concentrations between 20 $\mu M$ and 25 $\mu M$, suggests a complex interplay between CBD, cellular metabolism, and energy homeostasis. This observation is important due to the relationship between cAMP and cellular metabolism. cAMP is synthesized from ATP by adenylyl cyclases, directly linking cAMP levels to cellular energy status (Laudette et al. 2021). CBD was observed to have a broad range of effects on the cAMP metabolism. CBD can act as a strong inhibitor and negative allosteric modulator of CB1 signalling and partial/inverse agonist of CB2 signalling (Tham et al. 2019; Thomas et al. 2007). As both CB1 and CB2 are canonically coupled to $G_{\alpha i}$ subunits, cAMP concentrations should decrease upon activation, indicating that the observed cAMP increase is mediated by other mechanisms. Although it has been shown that CB2 can also couple to $G_{\alpha s}$ subunits and elicit a delayed increase in cAMP following prolonged incubation with $\mu M$ concentrations of CBD (Saroz et al. 2019), this delayed cAMP response may not be solely attributable to alternative CB2-$G_{\alpha s}$ signaling (Fig. 2). CBD has been demonstrated to activate Transient Receptor Potential Vanilloid receptors 1 and 2 (TRPV1/2) (Gochman et al. 2023; Qin et al. 2008; Starkus et al. 2019; Etemad et al. 2022; Anand et al. 2020; De Almeida And Devi 2020), resulting in an increase in intracellular $Ca^{2+}$ concentration. This elevation can subsequently activate calmodulin (CaM) signaling, leading to the activation of CaM-dependent adenylate cyclases (Neil et al. 1985). Furthermore, CBD functions as an inhibitor of the Equilibrative nucleoside transporter (ENT) 1 (Liou et al. 2008; Carrier et al. 2006), which leads to increased extracellular adenosine and subsequent activation of $G_{\alpha s}$-coupled adenosine receptors (e.g., A1, A2A) and their associated adenylate cyclases (De Almeida And Devi 2020; Carrier et al. 2006; Stollenwerk et al. 2021; Viczjan et al. 2022; Sheth et al. 2014. However, it has also been reported that CBD can act as a negative allosteric modulator of A2A, potentially attenuating the resulting cAMP increase (Raï et al. 2023; Sánchez-Fernández et al. 2024). An alternative, more indirect mechanism involves inhibition of cAMP-dependent phosphodiesterases such as PDE4, −7, or −8 (Delhaye And Bardoni 2021; Levy et al. 2011; Fertig And Baillie 2018), which slows cAMP degradation and thus indirectly elevates basal cAMP levels. While CBD is a potent inhibitor of the cGMP-specific PDE9, its binding to PDE4 has only been demonstrated so far (Ribaudo et al. 2023). Together, these mechanisms could explain the observed increase in cAMP. This shift may influence mitochondrial function, as cAMP is a key regulator of mitochondrial metabolism (Valsecchi et al. 2013). Elevated cAMP levels can enhance mitochondrial protein phosphorylation, a crucial mechanism for metabolic adaptation (Valsecchi et al. 2013). In mitochondria, cAMP signaling promotes post-translational regulation of oxidative phosphorylation, potentially stimulating ATP synthesis (Laudette et al. 2021). This could create a feedback loop where increased cAMP levels influence ATP generation, further affecting cellular energy balance. The cAMP/PKA pathway regulates glucose homeostasis at multiple levels, including glucose uptake, glycogen synthesis and breakdown, and gluconeogenesis (Yang And Yang 2016). Changes in cAMP levels induced by CBD may therefore have widespread effects on cellular metabolism. For instance, PKA activation by cAMP can phosphorylate and regulate key metabolic enzymes, potentially altering glycolysis, lipid metabolism, and other crucial metabolic pathways (Yang And Yang 2016). Furthermore, cAMP acts as a metabolic sensor through its interactions with AMP-activated protein kinase (AMPK), a central regulator of cellular energy homeostasis (Richani et al. 2019). Changes in cAMP levels can influence AMPK activity, potentially altering lipid and carbohydrate metabolism in response to CBD treatment. The observed anti-proliferative effects of CBD may be partially mediated through these cAMP-induced metabolic changes. Alterations in energy metabolism and mitochondrial function are known to influence cell cycle progression and apoptosis (Chen et al. 1998). The complex interactions between CBD, cAMP signaling, and cellular metabolism warrant further investigation to fully elucidate the mechanisms underlying CBD’s effects on HepG2 cells and its potential therapeutic applications.

### CBD alters LEDGF/p75 and caspase-3 expression in HepG2 cells

The opposite relationship between CBD concentration and the expression of caspase-3, LEDGF/p75, and GAPDH is particularly remarkable, especially when considering the role of LEDGF/p75 in cellular stress responses and cancer cell survival. LEDGF, also known as DFS70 or PSIP1, and in particular its longer splice variant LEDGF/p75, is a stress-response protein that plays a crucial role in protecting cells against various environmental stressors, including oxidative stress and cytotoxic drugs (Liedtke et al. 2021; Ortiz-Hernandez et al. 2024). In cancer cells, LEDGF/p75 has been shown to upregulate genes associated with oxidative stress responses, such as alpha B crystallin, involucrin, and peroxiredoxin (Kubo et al. 2002; Shin et al. 2008). The decrease in LEDGF/p75 expression with increasing CBD concentration aligns with CBD’s anti-proliferative effects, as LEDGF/p75 is known to promote cell survival and stress resistance (Liedtke et al. 2021; Ortiz-Hernandez et al. 2024). The downregulation of LEDGF/p75 by CBD could therefore compromise the cancer cells’ ability to cope with stress, potentially contributing to cell death. The observed decrease in caspase-3 expression initially appears to contradict the anti-proliferative effects of CBD, as caspases are key enzymes in the apoptotic process. However, this finding may indicate that CBD is triggering alternative cell death pathways or that the time-point of measurement does not capture the full dynamics of the apoptotic process. Recent research has demonstrated that CBD can induce various forms of programmed cell death in hepatocellular carcinoma (HCC) cells, including pyroptosis (Shangguan et al. 2021). Interestingly, LEDGF/p75 has been shown to be functionally inactivated by caspase-mediated cleavage during apoptosis (Ortiz-Hernandez et al. 2024). The decreased expression of both LEDGF and caspase-3 observed in our study might suggest that CBD is inducing a form of cell death that does not primarily rely on the classical apoptotic pathway. This is consistent with findings that LEDGF/p75 overexpression in cancer cells fails to protect against classical inducers of apoptosis such as staurosporine (Ortiz-Hernandez et al. 2024). Furthermore, LEDGF/p75 has been implicated in DNA repair processes, particularly in promoting homologous recombination (HR) at chromatin active sites (Ortiz-Hernandez et al. 2024). The downregulation of LEDGF/p75 by CBD could potentially impair the cancer cells’ ability to repair DNA damage, further contributing to cell death. This is particularly relevant given that CBD has been shown to induce DNA damage in cancer cells (Russo et al. 2019). The decrease in GAPDH expression may reflect overall changes in cellular metabolism induced by CBD treatment. GAPDH is not only a key glycolytic enzyme but also plays roles in various cellular processes, including apoptosis and oxidative stress response (Dando et al. 2013). Its downregulation indicates a shift in metabolic state or energy production in response to CBD treatment. Wang et al.’s study also revealed that CBD triggers mitochondrial stress and dysfunction by restraining the transcription of mitochondrial component proteins (Shangguan et al. 2021). They observed a decreased transcription of mitochondrial respiratory chain complex subunits and a dramatic decrease in oxygen consumption rate (OCR) after CBD treatment in HCC cells. This mitochondrial dysfunction was accompanied by membrane depolarization and loss of membrane potential.

### CBD and DNA integrity: reconciling contradictory findings

The available data (up to 2025) on the effect of CBD on DNA integrity is ambiguous: Whilst cell lines, such as HSC-3 and mononuclear leukocytes, show an increase in DNA damage and double strand breaks (Billi et al. 2025; Guler et al. 2020), others, such as H5V, HaCaT, A459, H1299 and H69 show no effect or a decrease (Bauer et al. 2024; Hamad And Olsen 2021; Li et al. 2022). For HepG2 cells, both an increase (Russo et al. 2019) and no effect have been reported (Štern et al. 2024). Our data has shown a significant, but slight decrease of both $\gamma$H2AX and 53BP1 foci for CBD concentrations between 5–50 $\mu M$ (Fig. 4). However, no significant dose-effect dependency was observable in this concentration range (Table 1). The apparent contradiction to the published studies indicating an increase in DNA damage might be explainable by the different experimental approach between our study and the study by Russo et al., where a COMET assay was used. The comet assay’s results can be influenced by several factors, including cell type, cell cycle stage, and specific experimental conditions (inter- and intra-laboratory variation) that may not always mimic in vivo situations accurately. Moreover, the comet assay might overlook certain types of DNA damage, such as double-strand breaks, which are more effectively detected using $\gamma$H2AX and 53BP1 foci analysis. Additionally, the presence of DNA repair enzymes can affect the comet assay’s results, potentially leading to an underestimation of DNA damage (Collins 2004; Olive and Banáth 2006; Kurashige et al. 2016; Cordelli et al. 2021).

### Further investigation of the influence of CBD on cancer

Our results indicate that CBD, within the tested concentration range, negatively affects HepG2 cell proliferation while having a slight reducing effect on DNA double-strand breaks. However, further research is necessary to comprehensively investigate CBD’s impact on human cancer. Expanding the tested concentration range from nanomolar to millimolar could help determine whether CBD’s influence is indeed dose-independent within the range of 5–50 $\mu M$ and elucidate the general dose-effect relationship. Additionally, testing a wider array of different exposure periods (e.g., 3 to 72 h) could help identify critical exposure times. To validate the observed effects of CBD on various tissues, cell culture studies should be supplemented with investigations using primary cells as well as both short- and long-term in vivo studies. As CBD is often used unregulated as a food supplement, the interaction between CBD and different common pharmaceuticals needs to be investigated to exclude negative interactions and improve consumer safety (Engeli et al. 2025).

## Conclusion

While our study demonstrates that CBD concentrations between 5–50 $\mu M$ have anti-proliferative effects on HepG2 cells and suggest a reduction in DNA double-strand breaks for short exposure times, it is essential to approach these findings with caution. The observed downregulation of LEDGF/p75 and Caspase-3, along with the reduction in $\gamma$H2AX and 53BP1 foci, indicates potential therapeutic benefits. However, these results contrast with previous studies that reported increased DNA damage under CBD treatment, underscoring the need for further investigation (Russo et al. 2019). The anti-proliferative effects of CBD observed in our study align with findings in other cancer cell lines (Mashabela And Kappo 2024), but the mechanisms underlying these effects remain incompletely understood. CBD was found to attenuate nuclear factor-$\upkappa$B (NF-$\upkappa$B) signaling and inhibit activation of the NLRP3 inflammasome, leading to a reduced secretion of pro-inflammatory cytokines like interleukin-1ß and tumour-necrosis-factor-$\upalpha$ (Liu et al. 2020; Rodrigues et al. 2024; Suryavanshi et al. 2021; Burstein 2015). Additionally, it was shown that CBD exhibits anti-oxidative properties and that it is able to sensitize cancer cells to chemotherapeutic agents like cisplatin (Ismail et al. (2025); Cherkasova et al. (2023); Li et al. (2022); Marini et al. (2024), but the long-term implications and safety profiles of such combinations are not fully elucidated. This duality of anti-proliferative/anti-cancerous and anti-inflammatory/anti-oxidative properties may lead to improved treatment efficacy of various cancer types with reduced side-effects. However, while it is crucial to acknowledge that CBD shows promise as an adjunct therapy, its interactions with various molecular targets and pathways can lead to unpredictable outcomes. Studies have highlighted both beneficial and harmful effects of CBD on DNA integrity, cell viability, and tumor progression. For instance, high concentrations of CBD have been linked to cytotoxic effects in certain cell types, raising concerns about potential genotoxicity and carcinogenicity.

In summary, while our data add to the accumulating evidence supporting CBD’s therapeutic potential, they also underscore the necessity for further research. Future studies should focus on elucidating the precise mechanisms of action, evaluating long-term effects, and ensuring the safe application of CBD in clinical settings.

## Supplementary Information

Supplementary Material 1.

## Figures and Tables

**Fig. 5:**
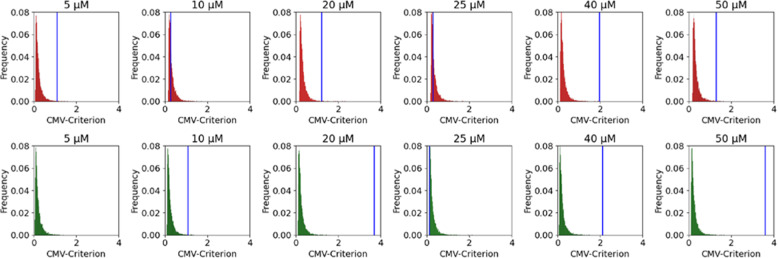
Histograms of the null distribution of the CMV criterion for $\gamma$H2AX (Top) and 53BP1 (Bottom). The calculated CMV criterion between the test group and the negative control is indicated by a blue line

**Fig. 6:**
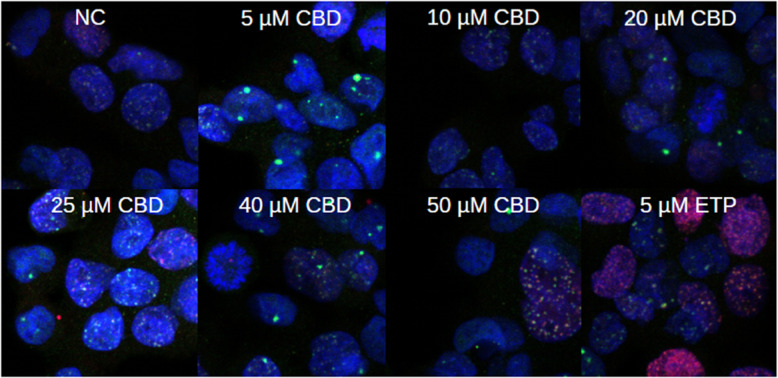
Indirect immunofluorescence images with $\gamma$H2AX and 53BP1 foci of HepG2 cells incubated with CBD (5 - 50 $\mu M$ in culture medium with 0.05 % ethanol as solvent; 5 $\mu M$ Etoposide (ETP) served as positive control). Cells were incubated for 3 h at 37 $^{\circ }$C in a 5 % $CO_2$ environment and directly fixated afterwards

## Data Availability

The data that support the findings of this study are available from the corresponding author upon reasonable request.

## Appendix

### 2 Buffers and solutions

**Table 2:** Laemmli Buffer (2x)

Reagent	Volume in mL	Final concentration
10 % (w/v) SDS	4	4 %
Glycerol	2	20 %
1 M Tris-CL	1.2	120 mM
$H_2O$	2.8	-
2 % bromophenol blue	0.1	0.02 %

**Table 3:** SDS Loading Buffer (5x)

Reagent	Final concentration
Bromophenol blue	0.25 %
Glycerol	50 %
SDS	10 %
Tris-CL (pH 6.8)	0.25 M
2-Mercaptoethanol	directly before usage; 5 %

**Table 4:** Running Buffer

Reagent	Final concentration
Tris/HCL (pH 8.5)	25 mM
Glycin	190 mM
SDS	0.1 %

**Table 5:** Transfer Buffer

Reagent	Final concentration
Tris/HCL	25 mM
Glycin	190 mM
Methanol	10 %

**Table 6:** Ponceau Red

Reagent	Final concentration
Ponceau S	2 %
Trichloroacetic acid	30 %
Sulfosalicylic acid	30 %

**Table 7:** TBS/TBST (pH 7.6)

Reagent	Final concentration
Trizma-HCL	24.23 g/L
NaCl	80.06 g/L
Tween 20	TBST only; 0.1 %

## Abbreviations

AMPK: AMP-activated protein kinase
ATP: Adenosine Triphosphate
CaM: Calmodulin
cAMP: Cyclic adenosine monophosphate
CBD: Cannabidiol
CB: Cannabinoid Receptor
DAPI: 4,6-diamidino-2-phenylindole
DMEM/F12: Dulbecco’s modified Eagle’s medium Nutrient Mixture F-12
DNA: Deoxyribonucleic acid
DSBs: DNA double-strand breaks
ECDF: Empirical Cumulative Distribution Function
ECL: Enhanced Chemiluminescence
ENT1: Equilibrative nucleoside transporter 1
ER: Endoplasmatic Reticulum
ETP: Etoposide
FCS: Fetal Calf Serum
FISH: Fluorescence *in situ* hybridization
GAPDH: Glyceraldehyde 3-phosphate dehydrogenase
GPCRs: G-protein coupled receptors
LEDGF/p75: Lens Epithelium-derived Growth Factor
MMPs: Matrix Metalloproteinases
n.s.: Not significant
OCR: Oxygen Consumption Rate
PBS: Phosphate-Buffered Saline
PDE: Phosphodiesterase
HCC: Hepatocellular Carcinoma Cells
PI: Propidium Iodide
PKA: Protein Kinase A
PPARs: Proliferator-activated Receptors
PVDF: Polyvinylidene Difluoride
THC: $$\Delta$$9-tetrahydrocannabinol
TRP: Transient Receptor Potential
VEGF: Vascular Endothelial Growth Factor
ROS: Reactive Oxygen Species
RT: Room Temperature
SCGE: Single-Cell Gel Electrophoresis
SDS-PAGE: Sodium Dodecyl Sulfate-Polyacrylamide Gel Electrophoresis
TRPV: Transient Receptor Potential Vanilloid
