# Supplementary figures and images for: Cannabidiol does not cause DNA double-strand breaks in a human liver-derived cell model
Source: J Cannabis Res. 2025 Dec 12;8:12. doi: 10.1186/s42238-025-00365-w (PMC12817681; doi:10.1186/s42238-025-00365-w)

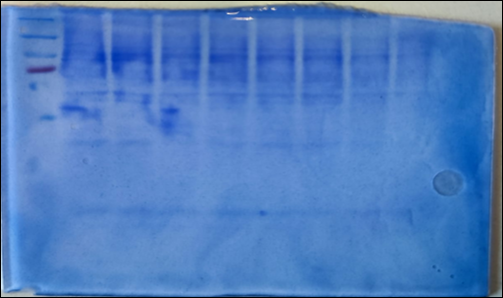

Supplement: Supplementary file 1 — Supplementary Material 1. [file 42238_2025_365_MOESM1_ESM.zip › WB3 - coomassie.tif]

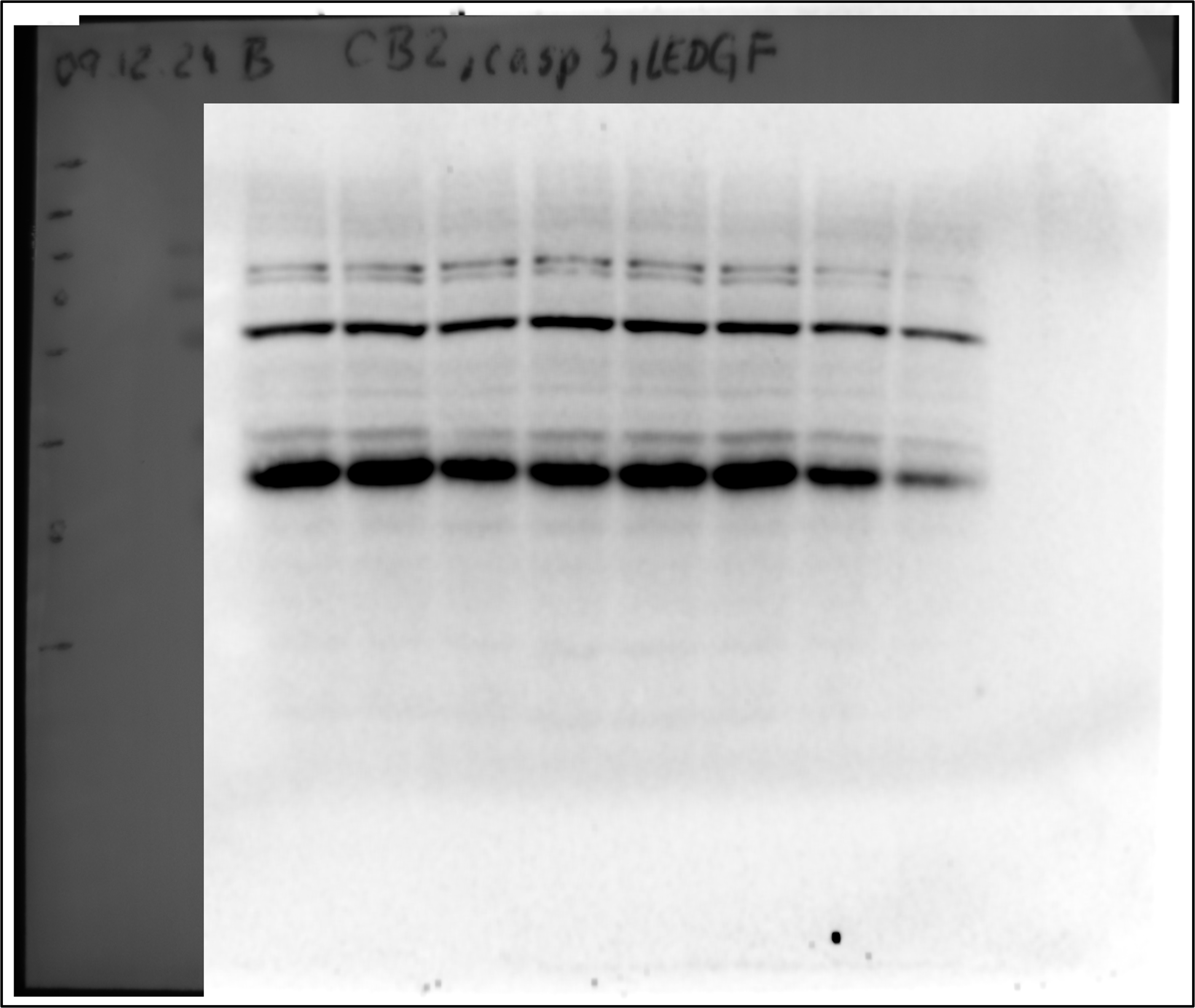

Supplement: Supplementary file 1 — Supplementary Material 1. [file 42238_2025_365_MOESM1_ESM.zip › WB3 - LEDGFp75.tif]

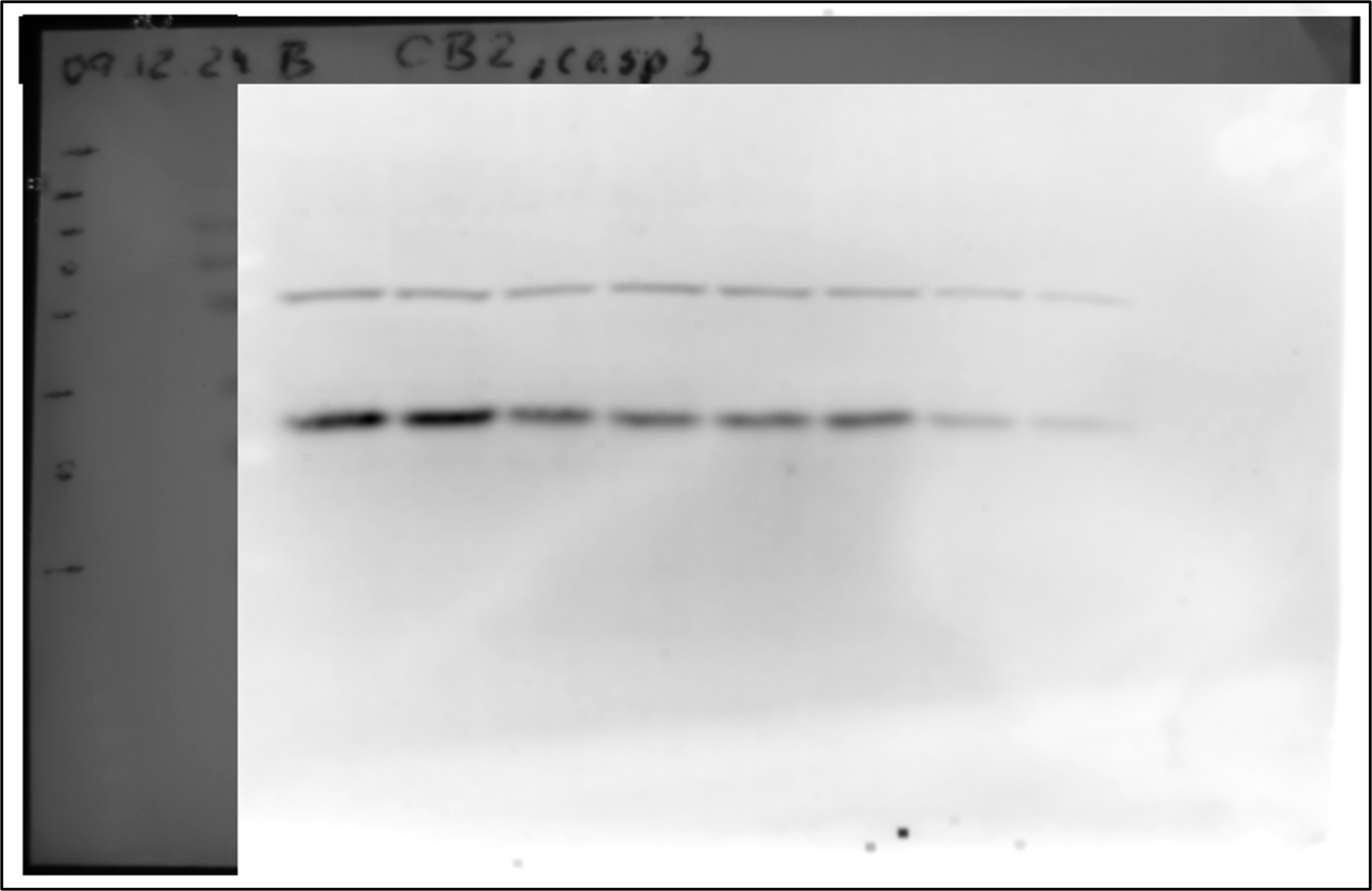

Supplement: Supplementary file 1 — Supplementary Material 1. [file 42238_2025_365_MOESM1_ESM.zip › WB3 - caspase-3.tif]

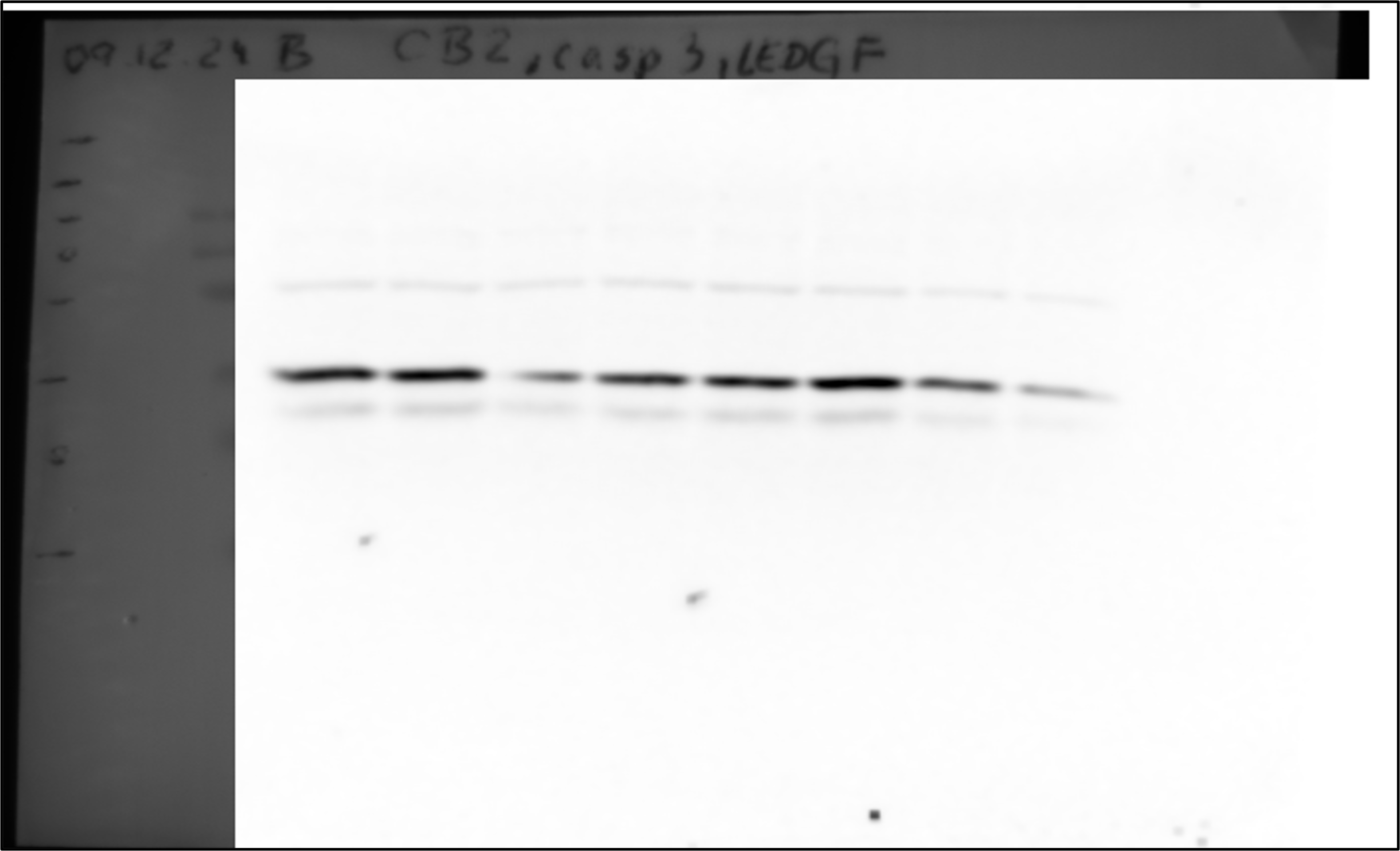

Supplement: Supplementary file 1 — Supplementary Material 1. [file 42238_2025_365_MOESM1_ESM.zip › WB3 - GAPDH.tif]

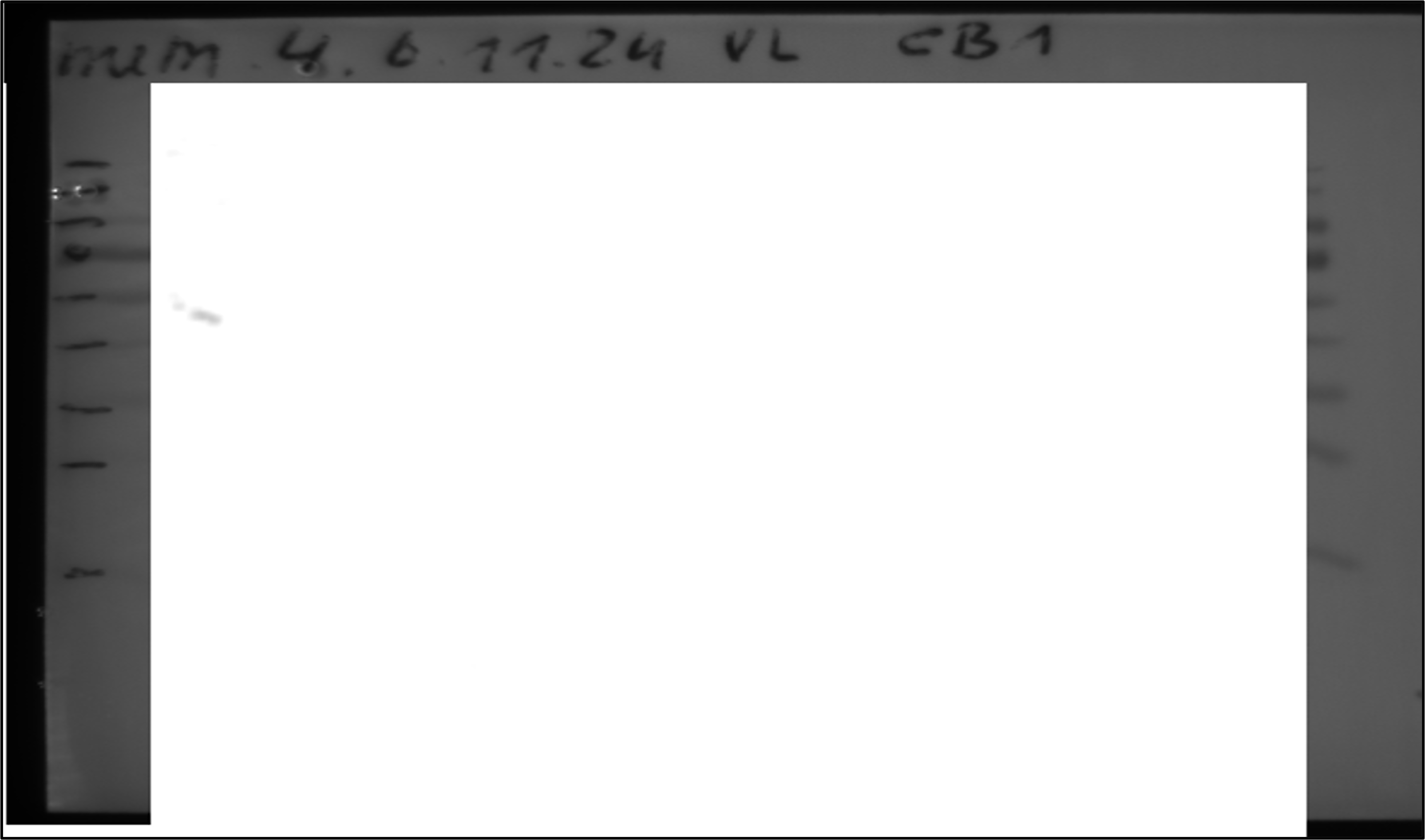

Supplement: Supplementary file 1 — Supplementary Material 1. [file 42238_2025_365_MOESM1_ESM.zip › WB3 - CB1.tif]

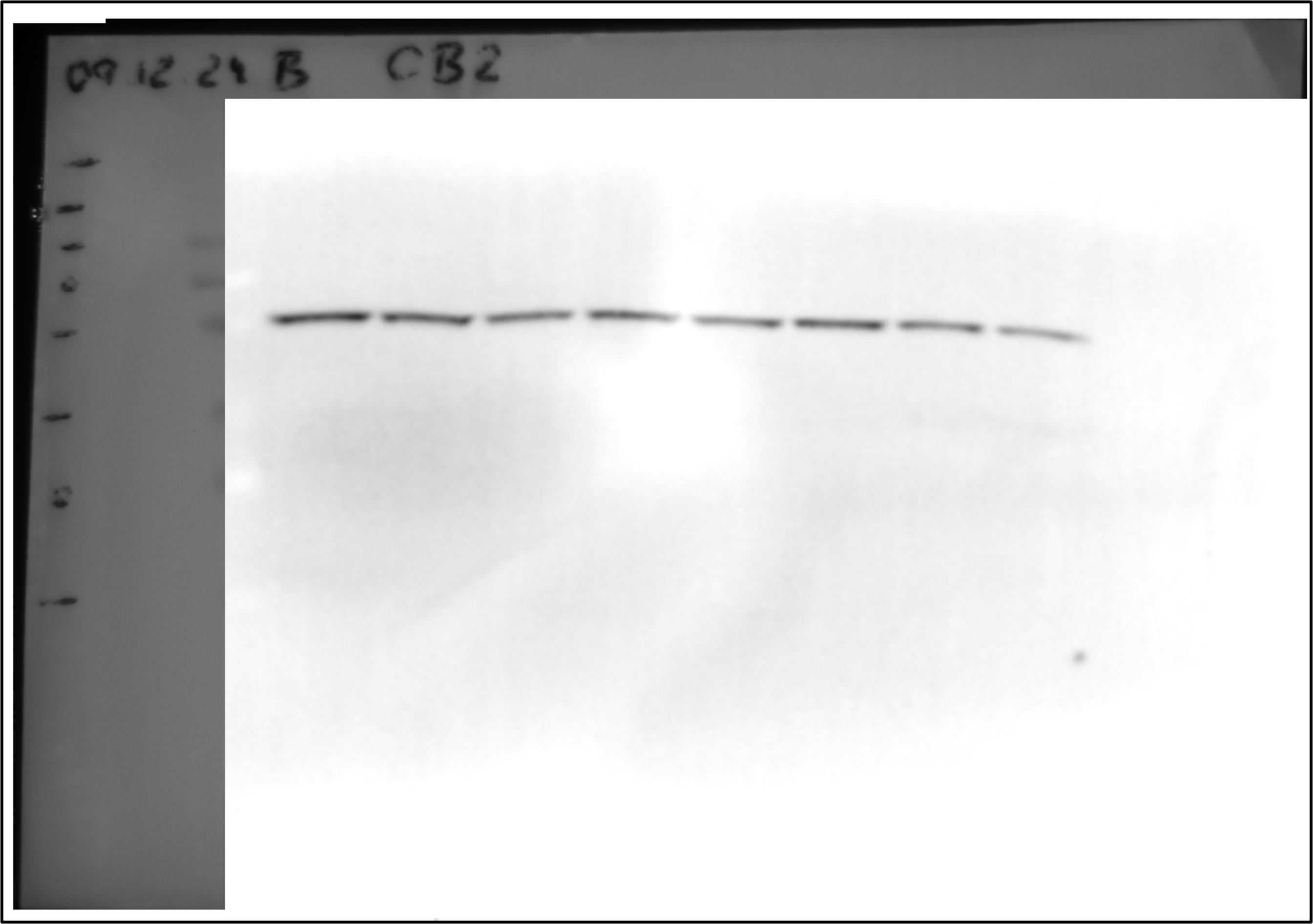

Supplement: Supplementary file 1 — Supplementary Material 1. [file 42238_2025_365_MOESM1_ESM.zip › WB3 - CB2.tif]

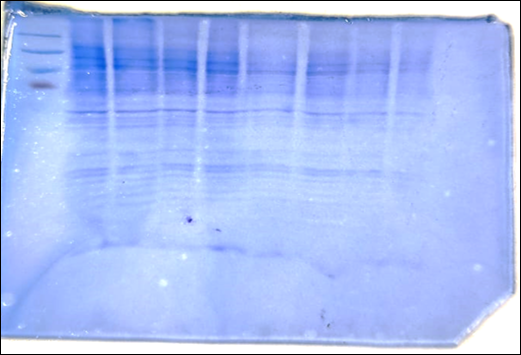

Supplement: Supplementary file 1 — Supplementary Material 1. [file 42238_2025_365_MOESM1_ESM.zip › WB2 - coomassie.tif]

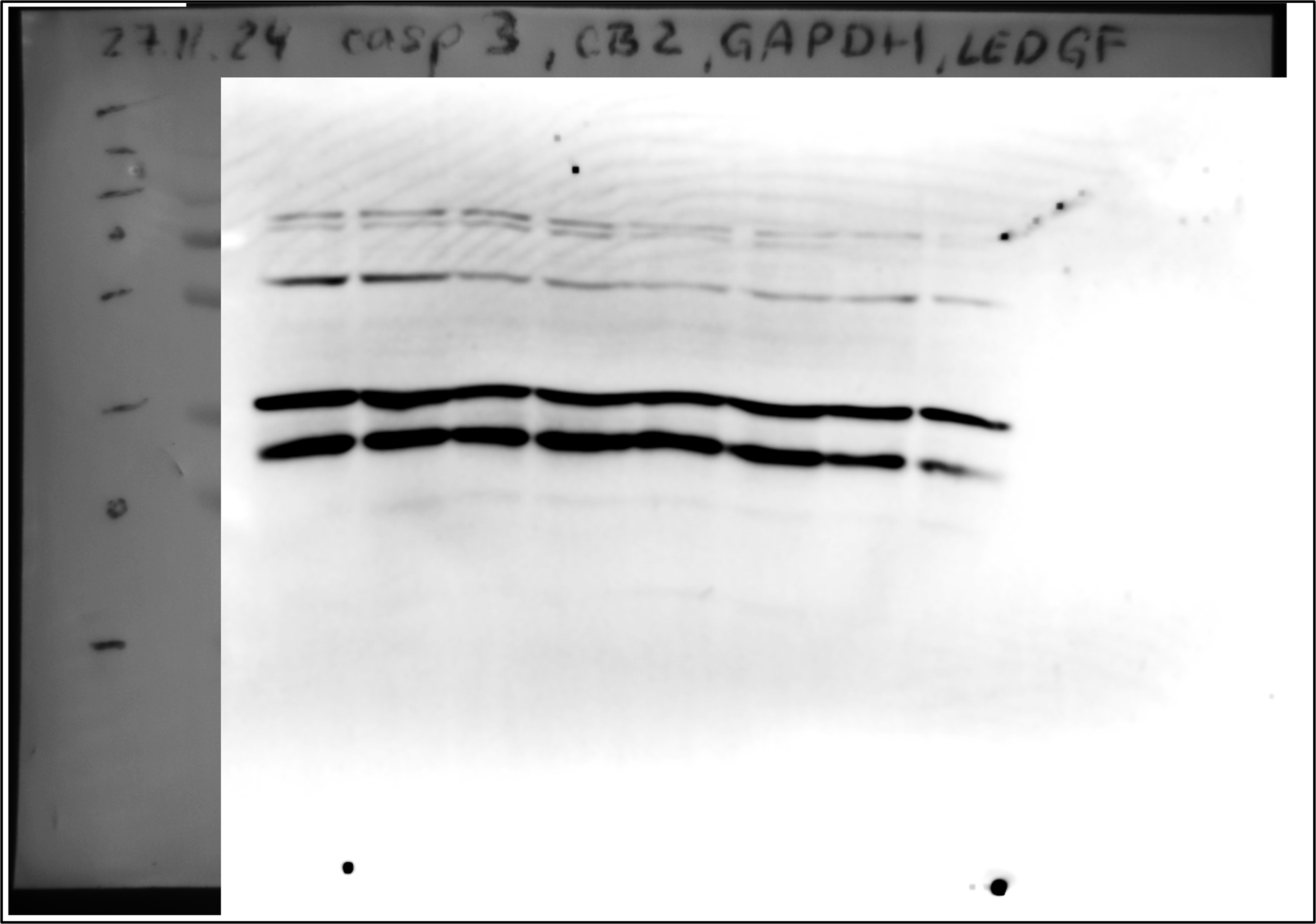

Supplement: Supplementary file 1 — Supplementary Material 1. [file 42238_2025_365_MOESM1_ESM.zip › WB2 - LEDGFp75.tif]

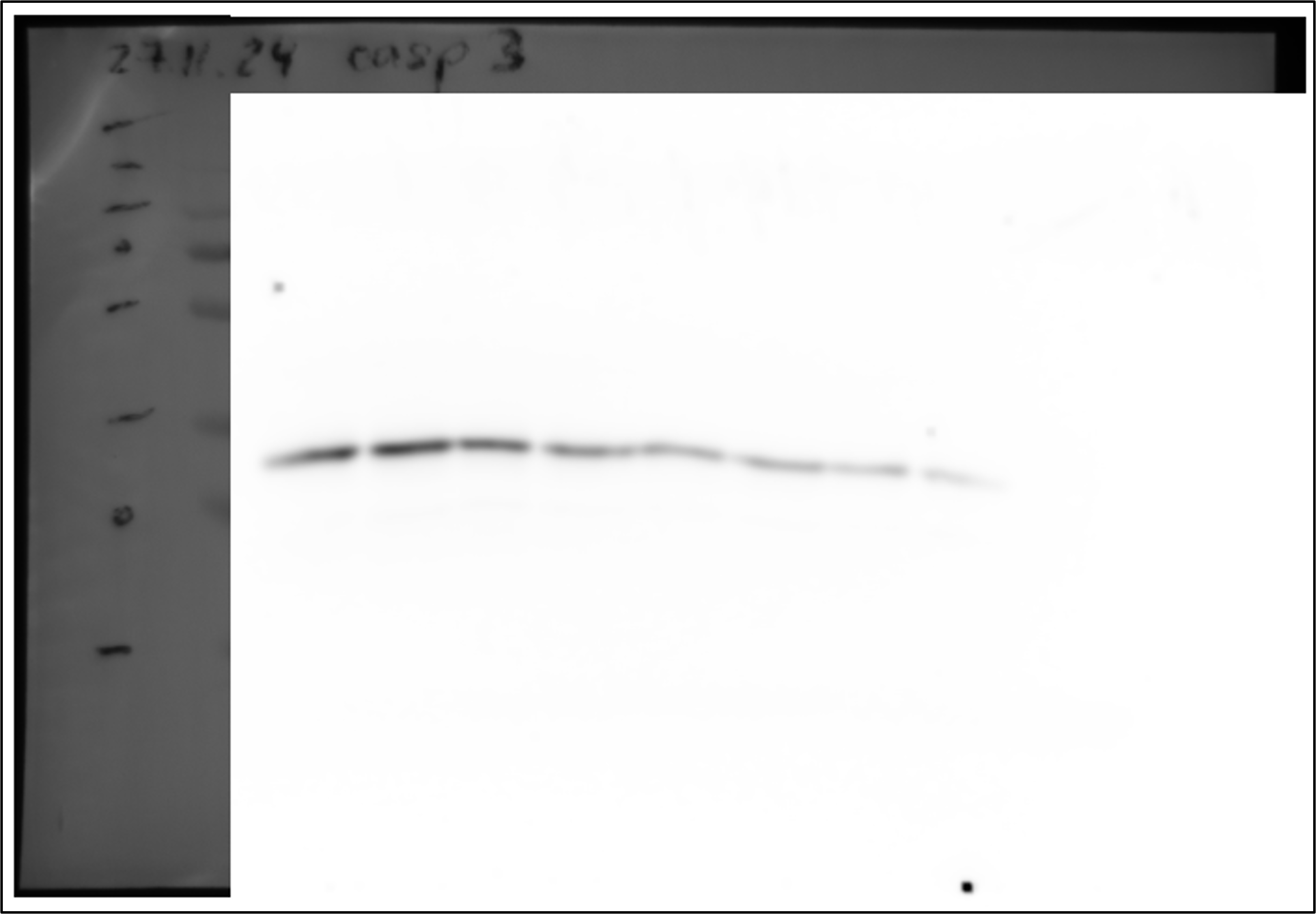

Supplement: Supplementary file 1 — Supplementary Material 1. [file 42238_2025_365_MOESM1_ESM.zip › WB2 - caspase-3.tif]

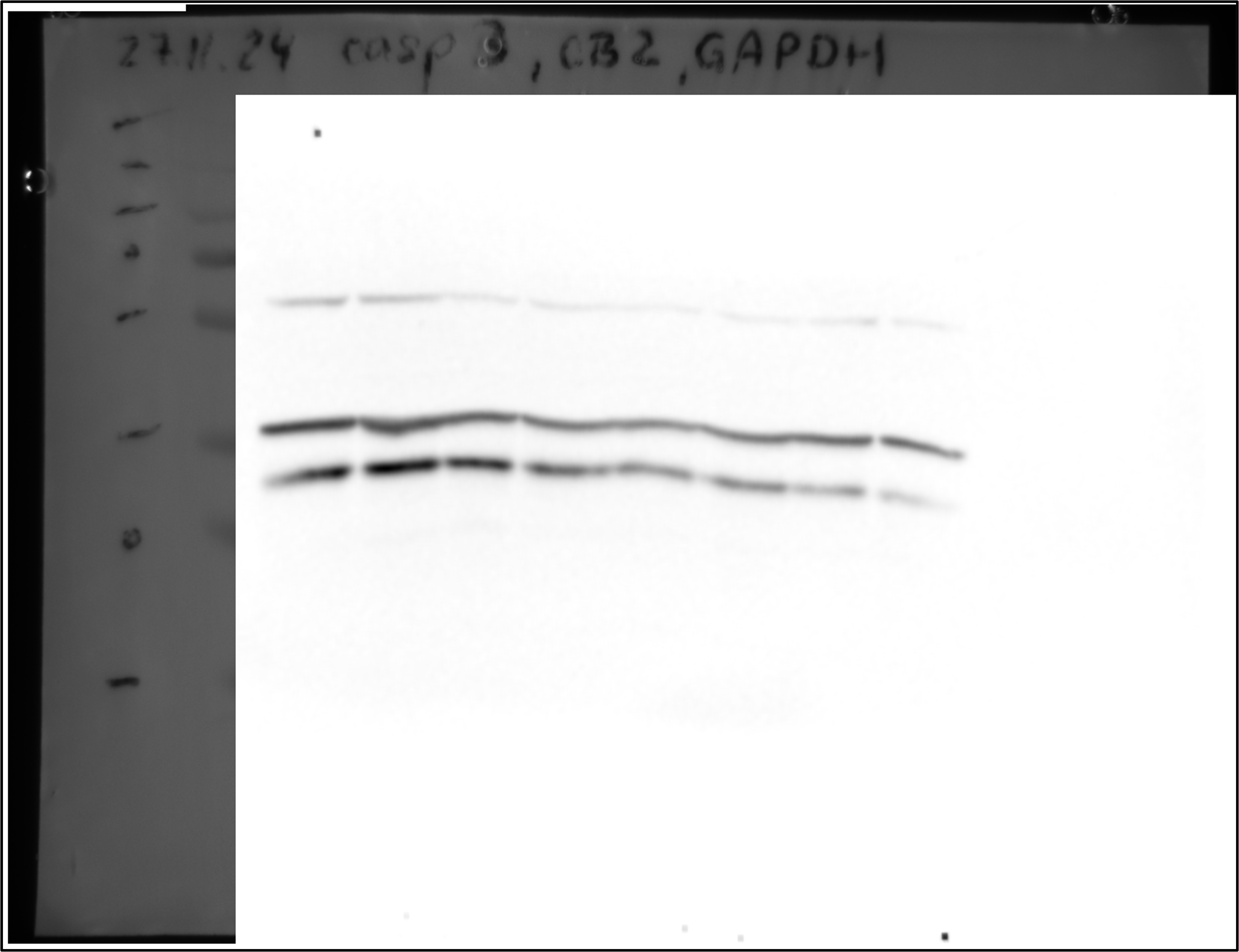

Supplement: Supplementary file 1 — Supplementary Material 1. [file 42238_2025_365_MOESM1_ESM.zip › WB2 - GAPDH.tif]

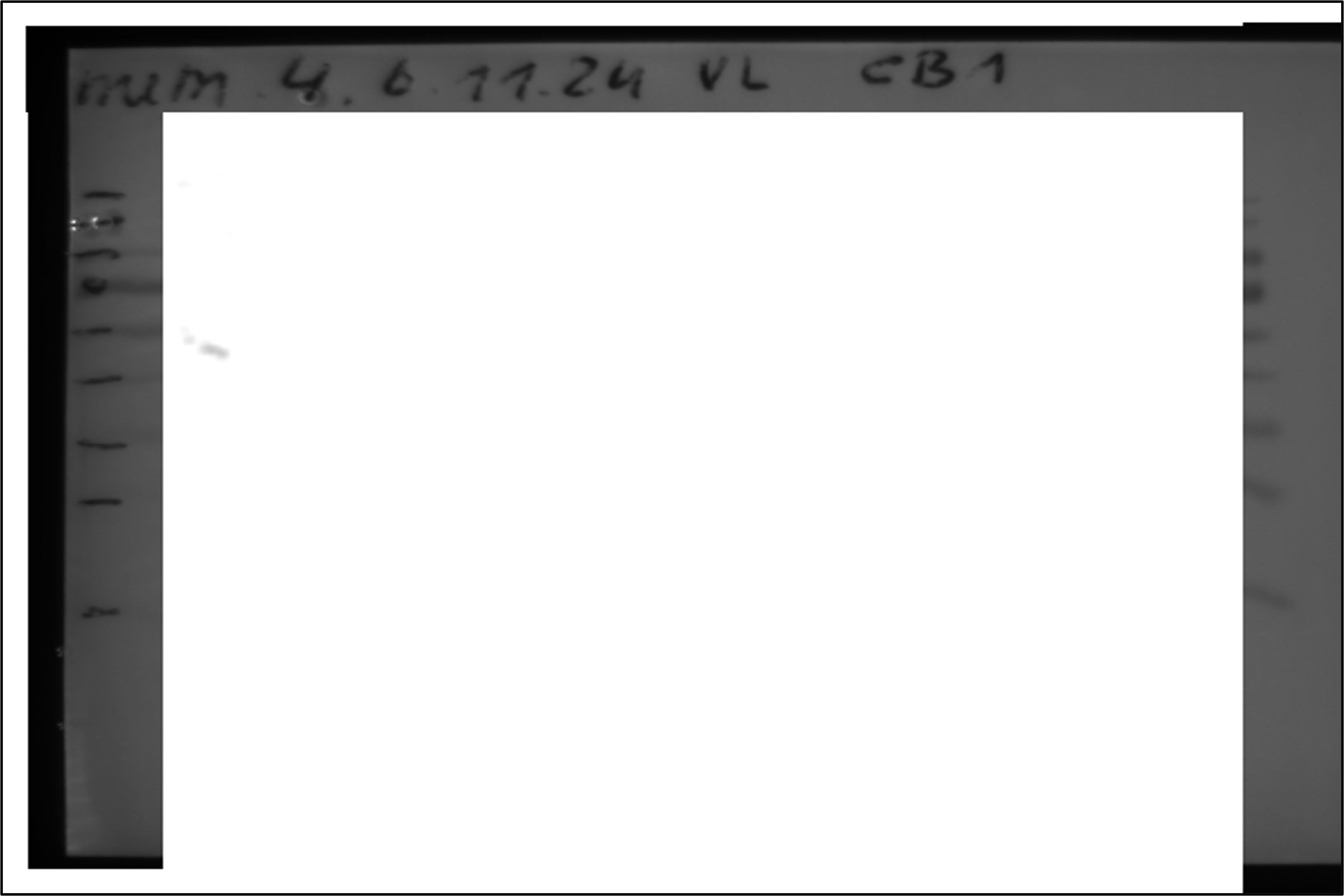

Supplement: Supplementary file 1 — Supplementary Material 1. [file 42238_2025_365_MOESM1_ESM.zip › WB2 - CB1.tif]

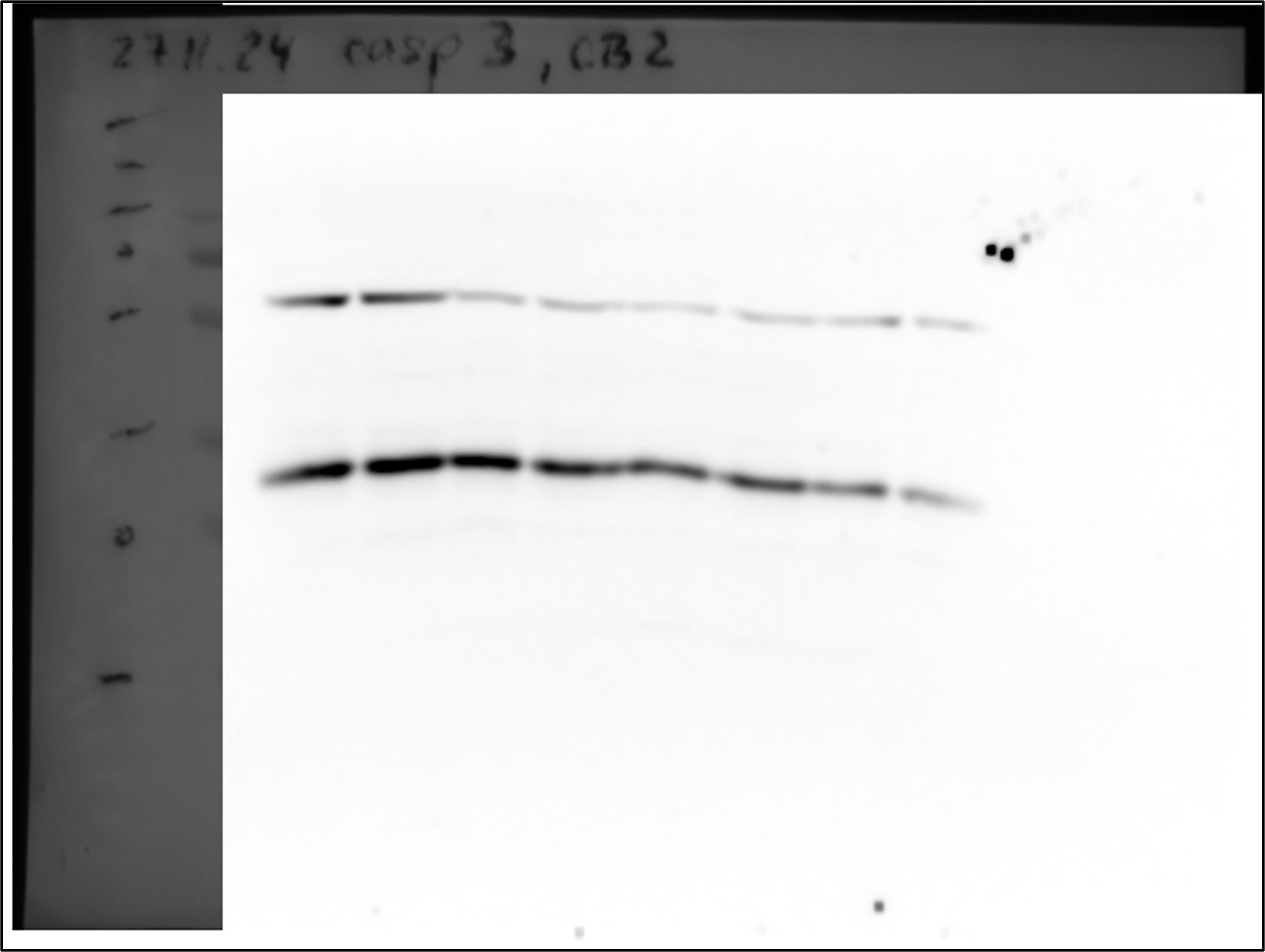

Supplement: Supplementary file 1 — Supplementary Material 1. [file 42238_2025_365_MOESM1_ESM.zip › WB2 - CB2.tif]

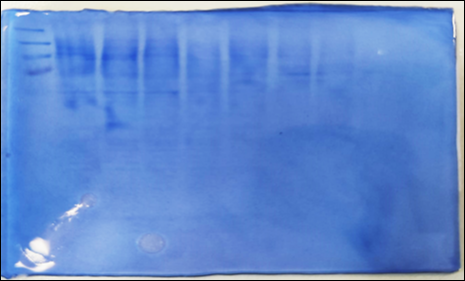

Supplement: Supplementary file 1 — Supplementary Material 1. [file 42238_2025_365_MOESM1_ESM.zip › WB1 - coomassie.tif]

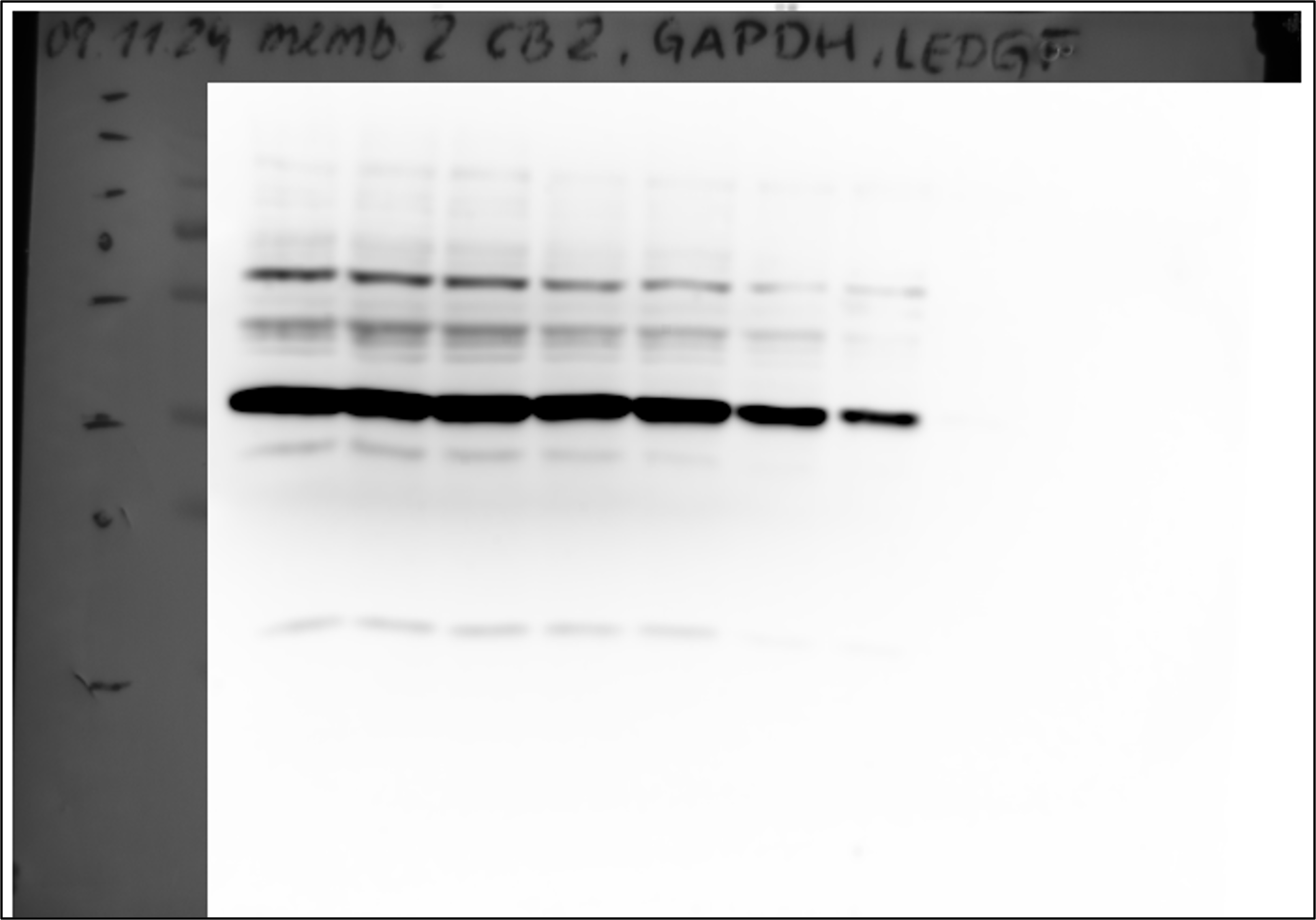

Supplement: Supplementary file 1 — Supplementary Material 1. [file 42238_2025_365_MOESM1_ESM.zip › WB1 - LEDGFp75.tif]

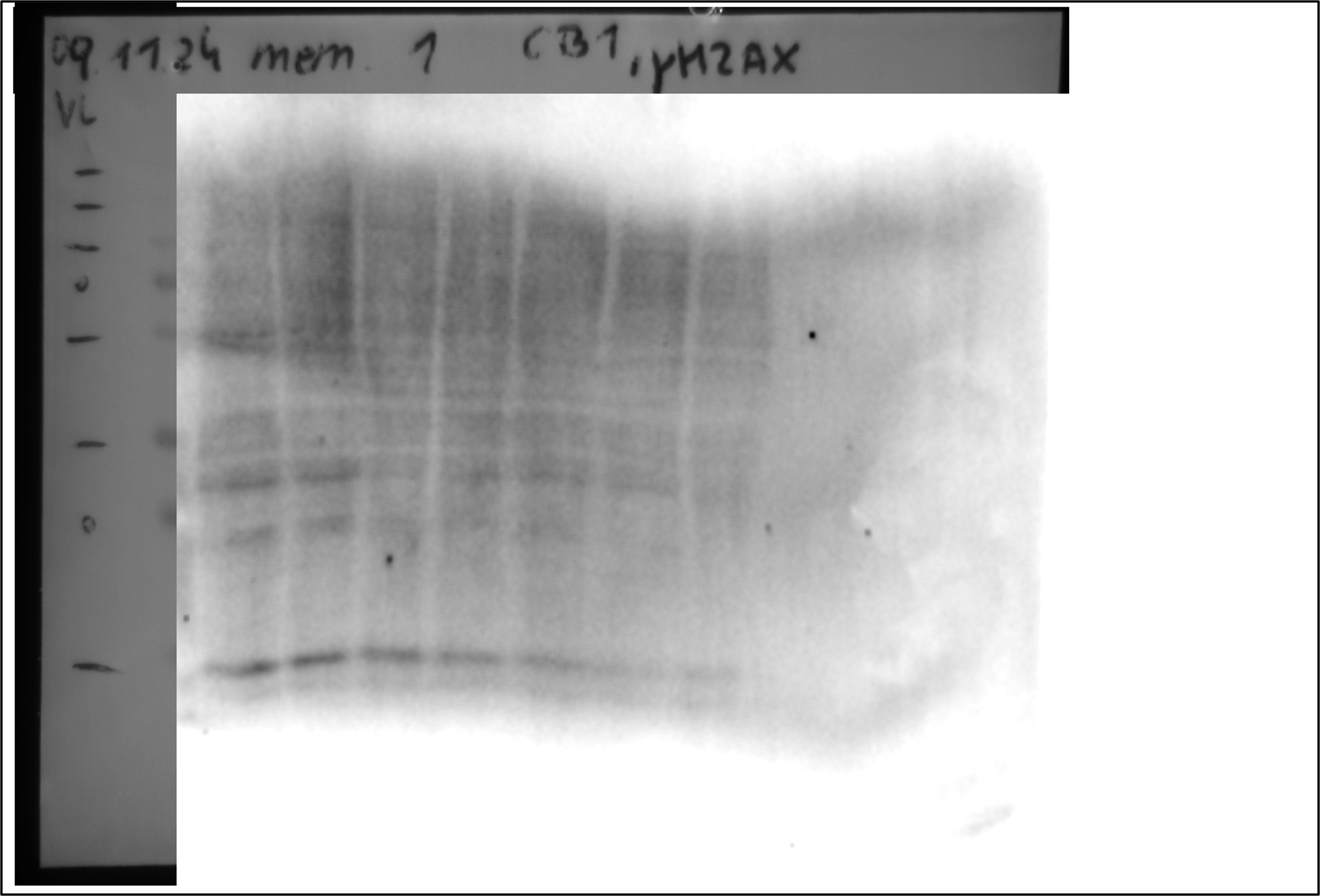

Supplement: Supplementary file 1 — Supplementary Material 1. [file 42238_2025_365_MOESM1_ESM.zip › WB1 - yH2AX.tif]

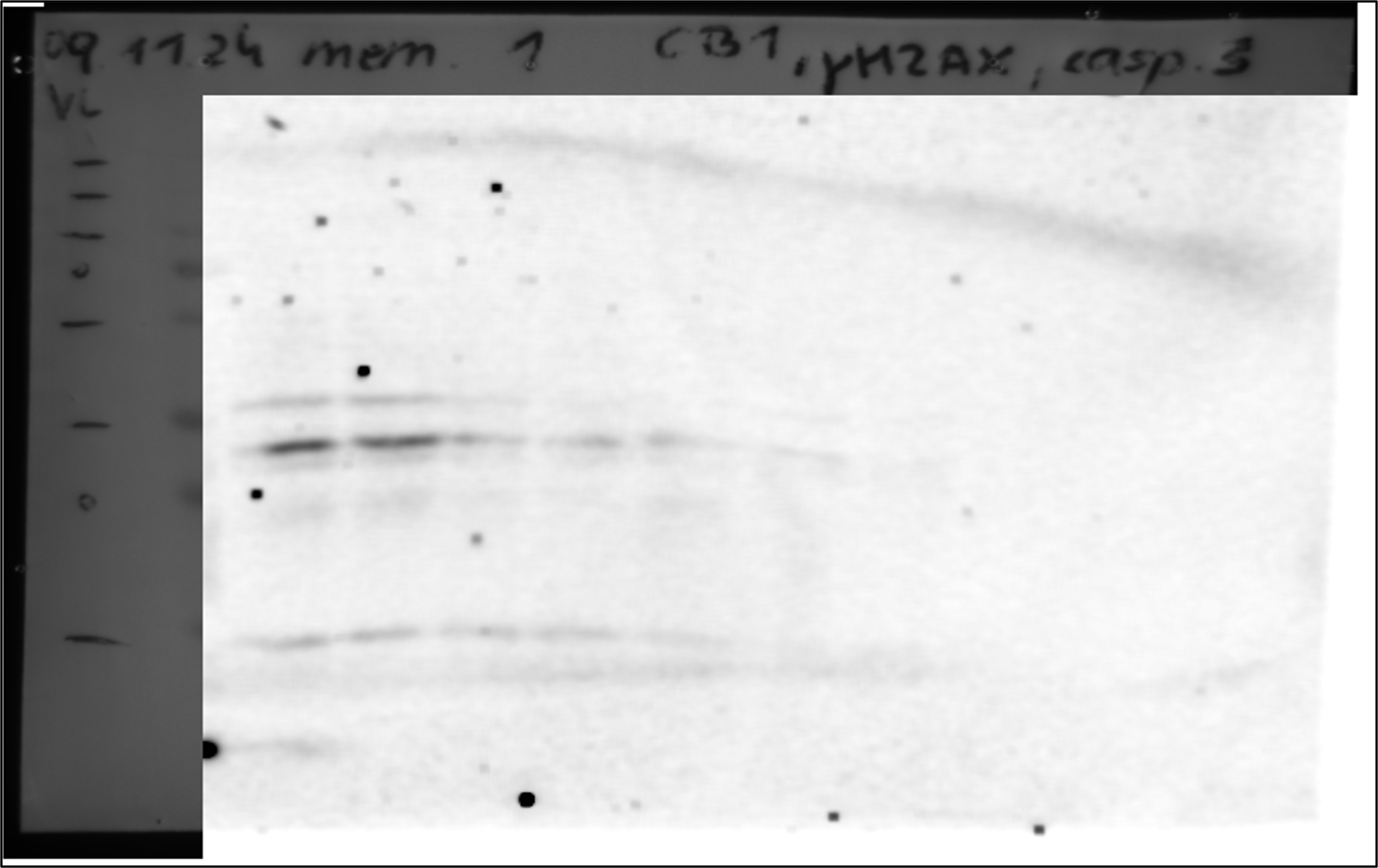

Supplement: Supplementary file 1 — Supplementary Material 1. [file 42238_2025_365_MOESM1_ESM.zip › WB1 - caspase-3.tif]

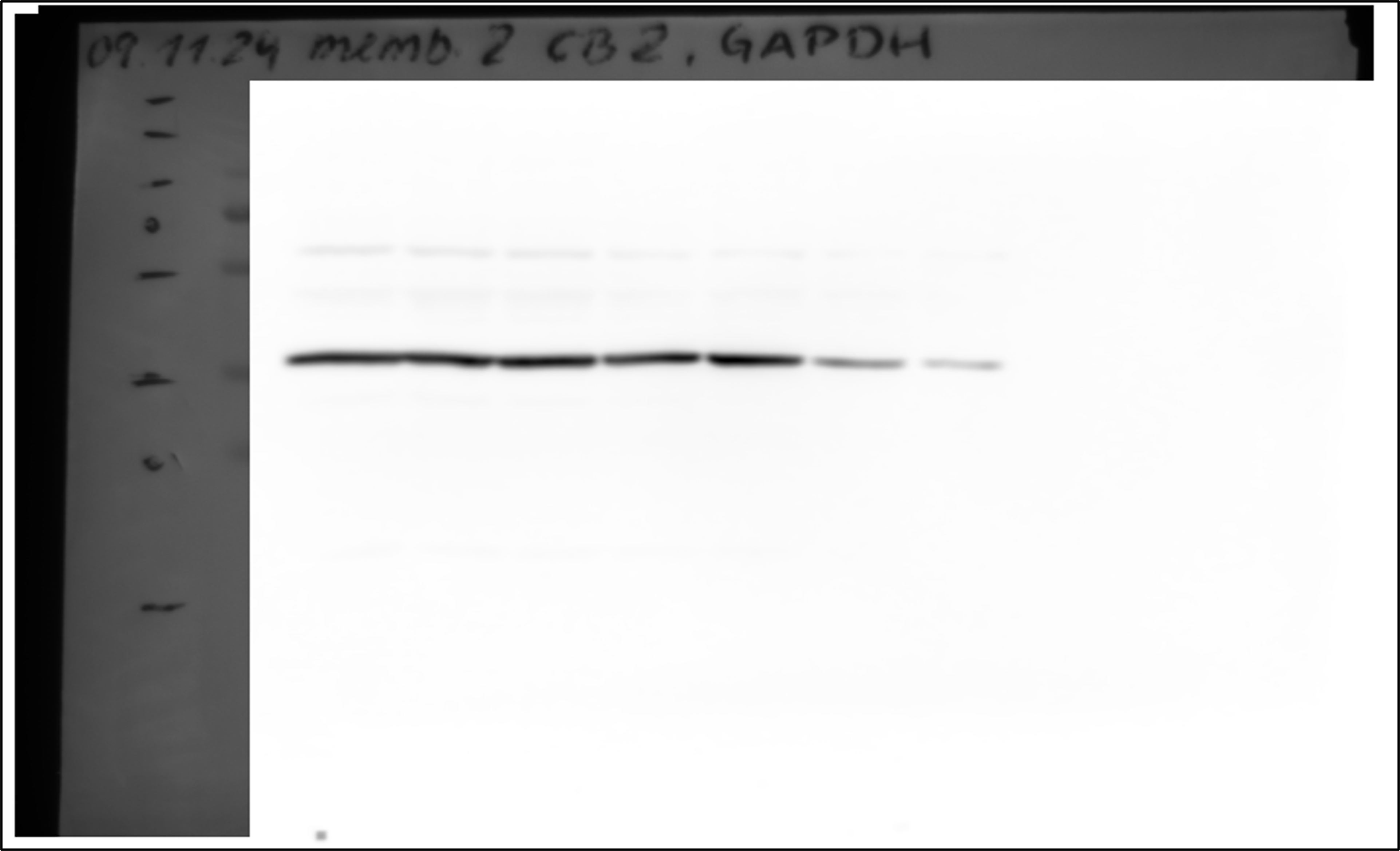

Supplement: Supplementary file 1 — Supplementary Material 1. [file 42238_2025_365_MOESM1_ESM.zip › WB1 - GAPDH.tif]

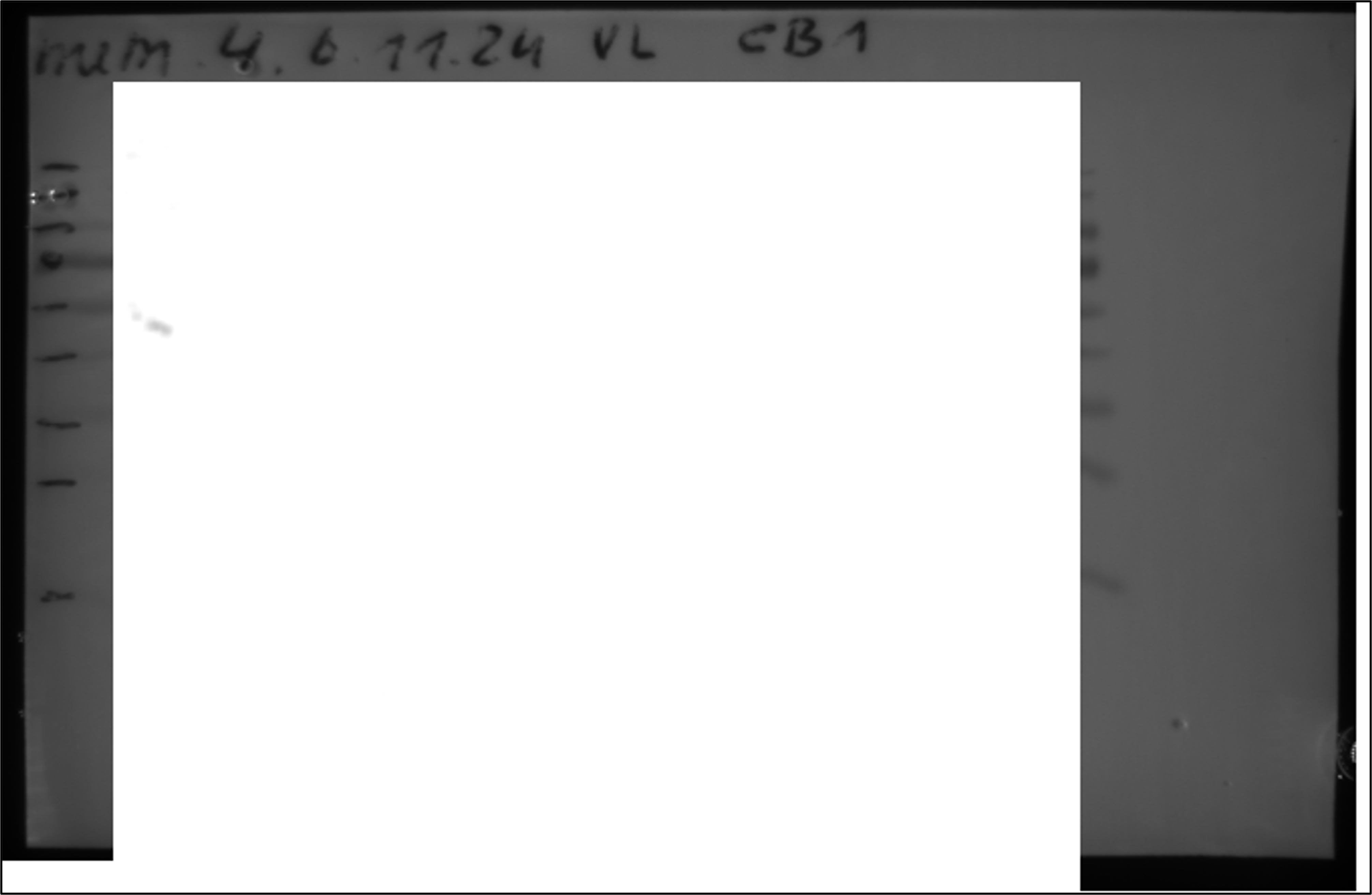

Supplement: Supplementary file 1 — Supplementary Material 1. [file 42238_2025_365_MOESM1_ESM.zip › WB1 - CB1.tif]

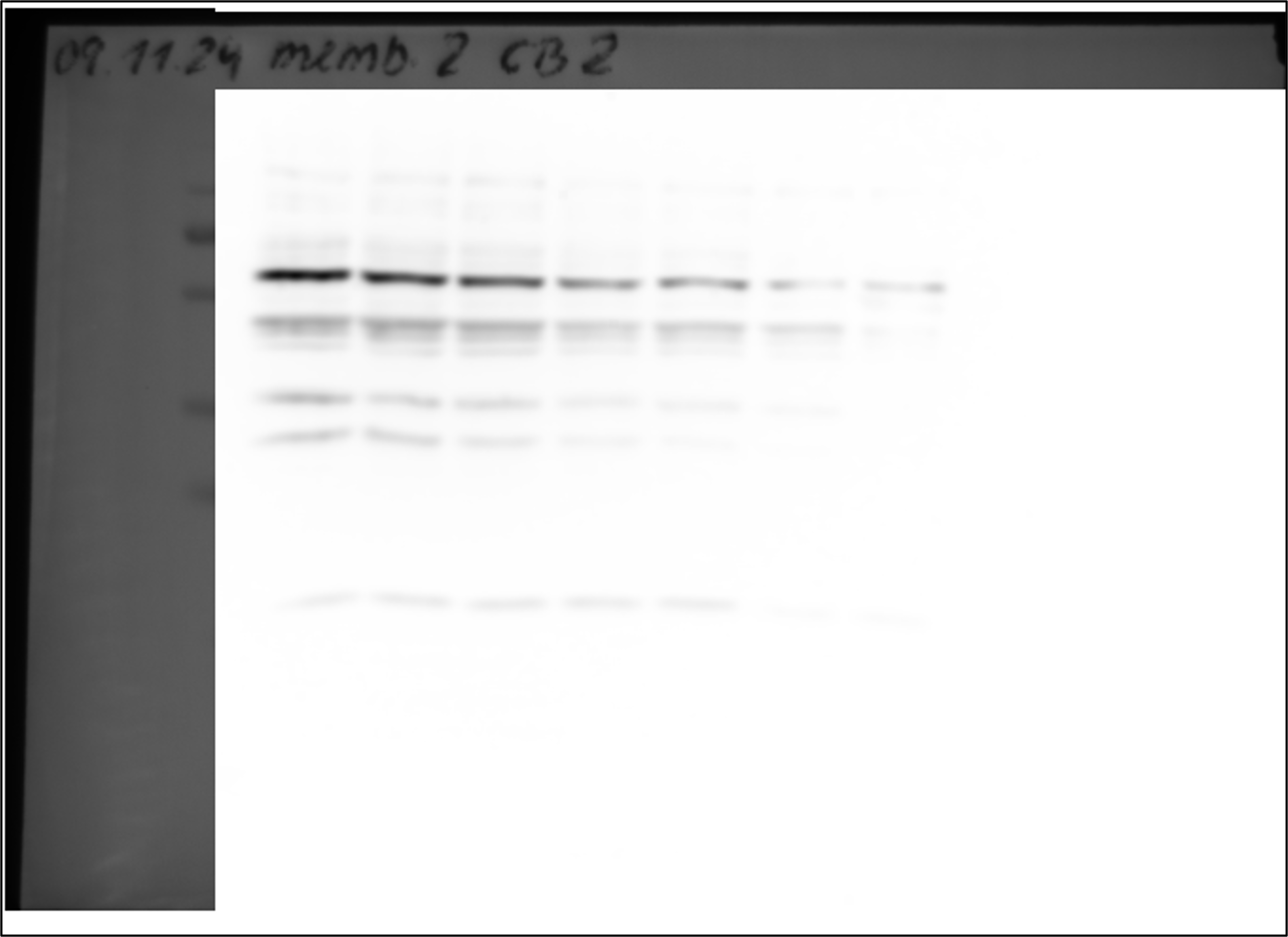

Supplement: Supplementary file 1 — Supplementary Material 1. [file 42238_2025_365_MOESM1_ESM.zip › WB1 - CB2.tif]
